# *Oct4* and *Hnf4α*-induced hepatic stem cells ameliorate chronic liver injury in liver fibrosis model

**DOI:** 10.1371/journal.pone.0221085

**Published:** 2019-08-12

**Authors:** Myung Rae Park, Man Sze Wong, Marcos J. Araúzo-Bravo, Hyunah Lee, Donggyu Nam, Soo Yong Park, Hong Dae Seo, Sang Min Lee, Hans Florian Zeilhofer, Holm Zaehres, Hans R. Schöler, Jeong Beom Kim

**Affiliations:** 1 Hans Schöler Stem Cell Research Center (HSSCRC), School of Life Sciences, Ulsan National Institute of Science and Technology (UNIST), Ulsan, South Korea; 2 Group of Computational Biology and Bioinformatics, Biodonostia Health Research Institute, San Sebastián, and IKERBASQUE, Basque Foundation for Science, Bilbao, Spain; 3 Department of Cranio-Maxillofacial Surgery, University Hospital Basel, Basel, Switzerland; 4 Department of Cell and Developmental Biology, Max Planck Institute for Molecular Biomedicine, Münster, Germany; Vrije Universiteit Brussel, BELGIUM

## Abstract

Direct conversion from fibroblasts to generate hepatocyte like-cells (iHeps) bypassing the pluripotent state has been described in previous reports as an attractive method acquiring hepatocytes for cell-based therapy. The limited proliferation of iHeps, however, has hampered it uses in cell-based therapy. Since hepatic stem cells (HepSCs) possess self-renewal and bipotency with the capacity to differentiate into both hepatocytes and cholangiocytes, they have therapeutic potential for treating liver disease. Here, we investigated the therapeutic effects of induced HepSCs (iHepSCs) on a carbon tetrachloride (CCl_4_)-induced liver fibrosis model. We demonstrate that *Oct4* and *Hnf4a* are sufficient to convert fibroblasts into expandable iHepSCs. Hepatocyte-like cells derived from iHepSCs (iHepSC-HEPs) exhibit the typical morphology of hepatocytes and hepatic functions, including glycogen storage, low-density lipoprotein (LDL) uptake, Indocyanine green (ICG) detoxification, drug metabolism, urea production, and albumin secretion. iHepSCs-derived cholangiocyte-like cells (iHepSC-CLCs) expressed cholangiocyte-specific markers and formed cysts and tubule-like structures with apical-basal polarity and secretory function in three-dimensional culture condition. Furthermore, iHepSCs showed anti-inflammatory and anti-fibrotic effects in CCl_4_-induced liver fibrosis. This study demonstrates that *Oct4* and *Hnf4α*-induced HepSCs show typical hepatic and biliary functionality *in vitro*. It also presents the therapeutic effect of iHepSCs in liver fibrosis. Therefore, directly converting iHepSCs from somatic cells may facilitate the development of patient-specific cell-based therapy for chronic liver damage.

## Introduction

Drug toxicity-induced chronic liver damage may lead to liver fibrosis that develops into liver cirrhosis and liver cancer [1, 2]. One of the histopathological aspects of liver fibrosis is an accumulation of extracellular matrix, such as collagen, originating from activated hepatic stellate cells. The phenomenon of collagen accumulation in liver fibrosis alters liver architectures and disrupts its functions [1, 2].

Previously, several studies have suggested that transplantation of hepatocytes or hepatoblasts derived from pluripotent stem cells (PSCs) show promising therapeutic effects in treating liver diseases [3–8]. However, the tumorigenicity of residual undifferentiated PSCs within the differentiated population is a major safety issue of using PSC-derived hepatic cells for clinical purposes [9]. Direct conversion technology, which generates desired cell-types from somatic cells by bypassing the pluripotent state during lineage conversion, has been considered an alternative method to generate hepatocytes, due to its relatively simple and fast procedure with possibly avoiding teratoma formation. Previous studies have demonstrated that generation of induced hepatocyte-like cells (iHeps) by various combinations of transcription factors show the therapeutic potential of iHeps in liver injury treatment [10–15]. Furthermore, expandable through several scale-up cultivation protocols, iHeps can be used as a source for drug discovery and screening [10–12]. Chen and colleague demonstrated that iHeps repopulate a decellularized liver disc [16]. In this study, the decellularized liver provides an optimal microenvironment for *in vitro* maturation of iHeps. However, these cells showed slow proliferation *in vitro*, which takes a long time to obtain sufficient numbers of the cell for cell-based treatment.

Hepatic stem cells (HepSCs) from liver tissue are self-renewing and bipotent stem cells that can differentiate into hepatocytes and cholangiocytes, which contribute to liver development and liver regeneration after injury [17–20]. HepSCs derived from the liver have considered as an alternative source for cell-based therapy. However, the resident HepSCs are rare in the adult liver [19], and the protocol for isolating HepSCs is still not well established [18, 19]. Oct4, known as a transcription factor required to maintain pluripotency, involved in hepatocyte proliferation and survival after 70% partial hepatectomy in the rat [21]. Zhao and colleagues demonstrated that *Oct4* maintains proliferative activity by inhibition of cyclin-dependent kinase 1 activity in PSC [22]. Our previous study reported that overexpression of *Oct4* and defined culture condition are sufficient to generate self-renewing and bipotent induced oligodendrocyte progenitor cells from fibroblasts [23]. We suppose that *Oct4* may play an essential role in transdifferentiation of iHepSCs and maintaining rapid cell proliferation in the iHepSCs.

Here, we suggest that the ectopic expression of *Oct4* and *Hnf4α* is sufficient to convert somatic cells into iHepSCs, which can be used for cell therapy to treat liver disease. The iHepSCs display self-renewal and bipotential characteristics *in vitro* and rescue liver dysfunction in a carbon tetrachloride (CCl_4_)-induced liver fibrosis model. Our study shows that iHepSCs may have therapeutic potential for chronic liver damage.

## Materials and methods

### Ethics statement

All animal experimental procedures were approved and conducted under a protocol (UNISTIACUC-16-24) by the Animal Care and Use Committee of Ulsan National Institute of Science and Technology (UNIST, Ulsan, South Korea).

### Isolation of mouse primary hepatocytes

All mice were obtained from Hyochang Science (Daegu, South Korea). Animal handling is in accordance with UNIST animal protection guideline. Primary hepatocytes were isolated from 6–week–old male C57BL/6J mouse by the two-step collagenase perfusion method as described previously [24]. In brief, mice were anesthetized with isoflurane. Livers were perfused in situ through the inferior cava vein, with Hanks’ balanced salt solution (Sigma) containing 50mM EGTA, 1M Glucose and 1% penicillin/streptomycin, followed by Hanks’ balanced salt solution containing 1M Cacl2, 0.025% collagenase (Sigma) until loss of its firm texture. The soft liver was transferred to a petri dish and dissociated cells with forceps then filtered by 70 μm cell strainer and centrifuged at 500 rpm for 5 min. The pellet was resuspended with Williams’ medium E (Thermo Fisher Scientific) supplemented with 1% penicillin/streptomycin and carefully overlay 13.5% Percoll solution then centrifuged at 1500 rpm for 8 min. After centrifugation, the pellet was washed three times and resuspended in Williams’ medium E supplemented with 1% penicillin/streptomycin and L-glutamine. Cell number and viability were assessed using trypan blue. Cells were plated on collagen-coated six-well plates and incubated for 2–3 hours at 37°C, 5% CO_2_ incubator and then used for the next experiment. For RNA extraction, the total RNA was isolated after incubation. For albumin and urea secretion assay, the medium was replaced with fresh medium. Next 24 hours, the medium was collected then conducted ELISA.

### Reprogramming vector production

Murine *Hnf4α* and *Oct4* cDNAs were amplified by polymerase chain reaction (PCR) with Phusion High-Fidelity DNA Polymerase (NEB, M05305) from plasmids. The plasmid containing *Hnf4α* was a gift from Atsushi Suzuki (Addgene #33002). *Hnf4α* and *Oct4* cDNAs were cloned into the lentiviral SFFV vector [25]. The lentiviral plasmid, packaging plasmid (PAX2, Addgene #12260) and envelope plasmid (VSV-G, Addgene #8454) were co-transfected into 293T cells using X-tremeGENE 9 DNA Transfection Reagent (Roche, 06365787001). After 48 hr, medium containing virus particles were harvested through the 0.45 μm filters for removing cell debris and concentrated by ultracentrifugation as described before [26].

### Generation of induced hepatic stem cells (iHepSCs)

Mouse embryonic fibroblasts (MEFs) were isolated from C57BL/6J mouse embryos at embryonic day 13.5 (E13.5) after removing the head and all internal organs. MEFs were maintained in DMEM supplemented with 10% Fetal Bovine Serum (FBS), 1% penicillin/streptomycin, L-glutamine, 2-Mercaptoethanol and MEM non-essential amino acid (All items purchased from Thermo Fisher Scientific). To generate of iHepSC from somatic cells, MEFs (1.0 x 10^4^ cells) were seeded on the gelatin-coated dish. On the next day, the MEFs were infected with lentivirus encoding *Hnf4α* and *Oct4* in MEF medium containing 6 μg/ml protamine sulfate. After 3 days, the MEF medium was switched to hepatocyte Culture Medium (HEP) medium (William’s E Medium supplemented with Primary Hepatocyte Maintenance Supplements). The culture medium was changed every 2 days. We conducted further characterization of iHepSCs at early (P3) and late (p20) passage.

### *In vitro* differentiation of bipotent iHepSCs

For the generation of hepatocyte, iHepSC were seeded on the collagen-coated dish and cultured in HEP medium supplemented with 10 ng/ml Oncostatin M (R&D system). For cholangiocyte differentiation, iHepSCs (0.5 x 10^4^ cells) were mixed with collagen gel and placed on a 4-well dish, as described previously [27]. The culture medium was changed every 2 days.

### *In vitro* functionality of iHepSC-HEP

For glycogen storage, PAS (Muto Pure Chemical) staining was performed following the manufacturer’s instructions. For low-density lipoprotein (LDL) uptake assays, DiI-ac-LDL (Biomedical Technologies) was added the culture medium (200 ng/ml) and incubated for 4 hr. After a wash step, the LDL uptake cells were imaged by fluorescent microscopy (Leica). For ICG detoxification, Indocyanine green (ICG, Sigma) was dissolved in DMSO to achieve 100 mg/ml stock concentration. Cells were cultured in the medium with 1mg/ml ICG for 60 min at 37°C and 5% CO_2_ incubator, followed by washing with PBS three times for uptake assay. These cells were incubated into the ICG-free culture medium for an additional six hr at 37°C and 5% CO_2_ incubator then washed with PBS three times, for release assay. The same spots on the plate were tracked throughout the whole process. To investigate the expression levels of cytochrome P450 (CYP) family genes, fibroblasts, iHepSCs, iHepSCs-HEP, and primary hepatocytes were placed at 60% confluence in HEP medium without dexamethasone supplement. CYP inducers, such as 50 μM 3-methylcholanthrene (3-MC) and 25 μM Rifampicin (Rif), were treated for 72 hours, and the fresh medium containing CYP inducers was replaced every day. Then the expression levels were analyzed by qPCR. Dimethyl sulfoxide (DMSO) was used as a control. For CYP P450 activity assay, the P450-Glo CYP1A2 assay system (Promega) was used according to a modified manufacturer’s protocol. The luminescence was measured by GLOMAX 96 micrometer luminometer (Promega). For quantification of albumin secretion and urea production, the amounts of albumin and urea in the culture media were analyzed using the Albumin ELISA Kit (Bethyl Laboratories) and Urea kit (BioAssay systems) according to the manufacturer’s instructions.

### Rhodamine 123 transport assay

To measure the activity of the membrane channel Multidrug Resistance Protein 1 (Mdr1), the cystic iHepSC-CLC were incubated with 10 μM verapamil (Sigma) for 30 min at 37°C and 5% CO_2_ incubator and the rhodamine assay was performed. Cystic iHepSC-CLCs were incubated with 100 μM rhodamine 123 (Sigma) for 5 min at 37°C and 5% CO_2_ incubator, then washed with PBS three times. Following the completion of each experiment, images were taken using a confocal microscope (Olympus). The fluorescence intensity in the lumen was normalized to the background measured in the surrounding external area.

### RT-PCR and quantitative real-time PCR (qRT-PCR)

The total RNA was isolated from fibroblasts, iHepSCs, iHepSCs-HEP, iHepSCs-CLC and primary hepatocytes using RNeasy Mini Kit (Qiagen) while total RNA from liver tissues and bile duct was extracted using TRIzol Reagent (Invitrogen). All mice were obtained from Hyochang Science (Daegu, South Korea). The adult liver tissue and bile duct were collected from 6-week-old male C57BL/6J mouse, and the embryonic day 14.5 (E14.5) fetal liver tissues were isolated from Pregnant C57BL/6J mouse as described previously [24, 28]. Total RNA (500 ng/mg) was used to synthesize cDNA with Omniscript Reverse Transcriptase (Qiagen) using oligo-dT primers. Reverse transcription polymerase chain reaction (RT-PCR) was performed using Taq polymerase (Invitrogen). Quantitative real-time PCR was performed on instrument LightCycler 480 using SYBR Green I Master (Roche). The experiments were performed in triplicate analysis and normalized to the housekeeping gene (*Gapdh*). Expression levels were compared using the comparative Ct method. The sequences of the primers are listed in S2 Table.

### Immunocytochemistry (ICC) analysis

iHepSCs or iHepSC derivatives were fixed with 4% paraformaldehyde (PFA) for 10 min and permeabilized with 0.1% Triton X-100 for 10 min at room temperature (RT). The cells were incubated with blocking solution (4% FBS in DPBS) for 30 min. Cells were then incubated with primary antibodies for 60 min at RT. Next, the cells were washed with DPBST (0.05% Tween20 in DPBS). The secondary antibodies were incubated in the dark for 60 min. Nuclei were stained with DAPI (Invitrogen). Cells were imaged by fluorescent microscopy (Leica). The antibodies used for analysis are listed in S3 Table. Appropriate positive and isotype control antibodies were used in all immunostaining experiments. The images of these experiments shown in S12 and S13 Figs.

### Growth curve and mean doubling time

Cell proliferation assay was performed as described previously [23]. iHepSC clones (1 × 10^4^ cells) were plated onto 12–well plates and cultivated for 10 days. The cells were collected from triplicate wells and manually counted each day using a hemocytometer (Marienfeld). The average cell numbers on each day were plotted, and the mean doubling time was calculated based on the growth curve.

### Genotyping of iHepSC

Genotyping was performed on genomic DNA isolated from iHepSC using the protocol as previously mentioned [29, 30]. Briefly, iHepSCs lysed by digestion at 55°C in extraction buffer (100 mM EDTA, 50 mM TRIS-HCl, 100 mM NaCl, 1% SDS, and 1.0 mg/ml proteinase K). DNA was precipitated by adding isopropanol, washed twice with 70% ethanol (v/v), and resuspended in Tris-EDTA buffer (pH 8.0). Proteinase K After heat-inactivation for 15 min, PCR was carried out with the following conditions: 94°C 30 s (1 cycle); 94°C 10 s, 57°C 30 s, 72°C 30 s (40 cycles); 72°C 5 min by using vector and CDS specific primer. The primer is listed in S2 Table.

### Microarray analysis

Global gene expression of the following cell populations was profiled by microarray analysis: fibroblasts (MEF), primary hepatocyte (pHep), iHepSC passage 3 (iHepSC-P3) and passage 20 (iHepSC-P20). We compared these samples with previously published data for wild type hepatic stem cells (wtHepSC, Dlk-positive hepatoblasts were sorted by magnetic-activated cell sorting (MACS) from isolated E14.5 mouse liver tissue) [28]. Total RNA of fibroblasts, iHepSC clones (P3, 20) and primary hepatocytes were isolated using an RNeasy mini kit (Qiagen) according to the manufacturer’s instructions. The samples were hybridized to an Affymetrix Mouse 430 2.0 array. Normalization was performed with the Robust Multiarray Analysis (RMA) algorithm [31]. Data processing and graphic producing were performed with in-house developed functions in MATLAB. The hierarchical clustering of genes and samples was performed with the one minus correlation metric and the unweighted average distance (UPGMA) linkage method. Differentially expressed genes were analyzed by comparing gene expression of iHepSC clones, both iHepSC‒P3 and iHepSC‒P20, relative to fibroblasts. Among the highly upregulated genes (˃1.2 fold), genes that are related to hepatic function were screened according to Gene Ontology (GO) term enrichment profiling. Microarray data from the wtHepSCs were downloaded from the GEO database (data accessible at NCBI GEO database [28], accession GSE17462). The data discussed in this publication have been deposited in NCBI’s Gene Expression Omnibus [32] and are accessible through GEO Series accession number GSE106347 (http://www.ncbi.nlm.nih.gov/geo/query/acc.cgi?acc=GSE106347).

### Transplantation of iHepSCs into mouse liver failure model

All mice were housed in a 12 hr light/dark cycle with free access to water and food. The experimental procedures were carried out in accordance with the approved guidelines, and all protocols were approved by the Animal Care and Use Committee of Ulsan National Institute of Science and Technology (Ulsan, South Korea). For liver fibrosis model, 4 μl/g body weight Carbon tetrachloride (CCl_4_, Sigma) diluted 1:10 in corn oil (Sigma) were intraperitoneally injected into 6-week-old male C57BL/6J mice (n = 12) twice a week for 7 weeks as previously described [33]. iHepSCs (2 x 10^6^ cells/mouse) that were transduced with GFP vector before transplantation were dissociated by 0.05% trypsin-EDTA and transplanted by intrasplenic injection to liver under anesthesia. To localize transplanted iHepSCs, the livers were harvested at day 3 after cell transplantation. Mice were subsequently sacrificed at the indicated times after a one-week washout to eliminate acute effects of CCl_4_. At the end of the experiment period, plasma was collected from each mouse and stored at -80°C. Liver tissues from each mouse were excised and immediately snap-frozen in liquid nitrogen for further RNA analysis. A portion of the fresh liver was preserved in 4% PFA for histopathological analysis.

### Histological analysis

The liver tissues were fixed overnight in 4% PFA, embedded in paraffin or O.C.T compound (Leica) and sectioned sequentially at 8–15 μm. The sections were permeabilized with 0.2% Triton X–100 and blocked with CAS–Block solution (Invitrogen) to prevent non–specific binding. For immunofluorescence staining analysis, the sections were incubated with primary antibodies diluted in CAS–Block solution overnight at 4°C. After the primary antibody incubation, the sections were washed with DPBST three times for 10 min each. The following secondary antibodies were diluted in PBS and applied for 60 min. Images were visualized by fluorescence microscopy (Leica). The primary antibodies used for analysis are listed in S3 Table. The nucleus was stained with hematoxylin (Sigma) and blued in 0.2% ammonium hydroxide. Tissue was then placed in Eosin Y solution (Sigma) and followed by several washings and dehydrating. Slides were mounted with a mounting solution (Leica).

### Quantitative analysis of liver fibrosis

For quantification of the fibrotic liver tissue, the paraffin sections were stained with picrosirius red (Abcam) according to the manufacturer’s instructions. Images were obtained using a light microscope (Olympus) equipped with a CCD camera. The areas of staining were quantitated using the software Image J 1.51J (National Institutes of Health, Bethesda, MD) as described previously [34, 35]. Briefly, the red area, considered the fibrotic area, was assessed by computer-assisted image analysis with Image J at a magnification of ×100. The mean value of 3 randomly selected areas per sample was used as the expressed percent area of fibrosis.

### Assessment of liver function

Blood serum samples were taken for assessment and the amount of ALT (Alanine transaminase) was estimated by using the model of 7020 clinical analyzer (Hitachi).

### Teratoma formation assay

Teratoma formation assay was performed as described previously [29, 30]. iHepSCs (2 x 10^6^ cells/mouse) were injected subcutaneously of the dorsal flank of 5 athymic nude mice. 12 weeks after the injection, mice were sacrificed for analysis of teratoma formation. The experimental procedures were approved by the Animal Care and Use Committee at Ulsan National Institute of Science and Technology (Ulsan, South Korea).

### Statistical analysis

Data are reported as mean values from at least three replicates. Error bars denote standard deviation (SD). Statistical significance was evaluated with unpaired two-tailed Student’s t-test using Microsoft Excel. (*p < 0.05).

## Result

### Generation of induced hepatic stem cells (iHepSCs) by *Oct4* and *Hnf4α*

**Fig 1:**
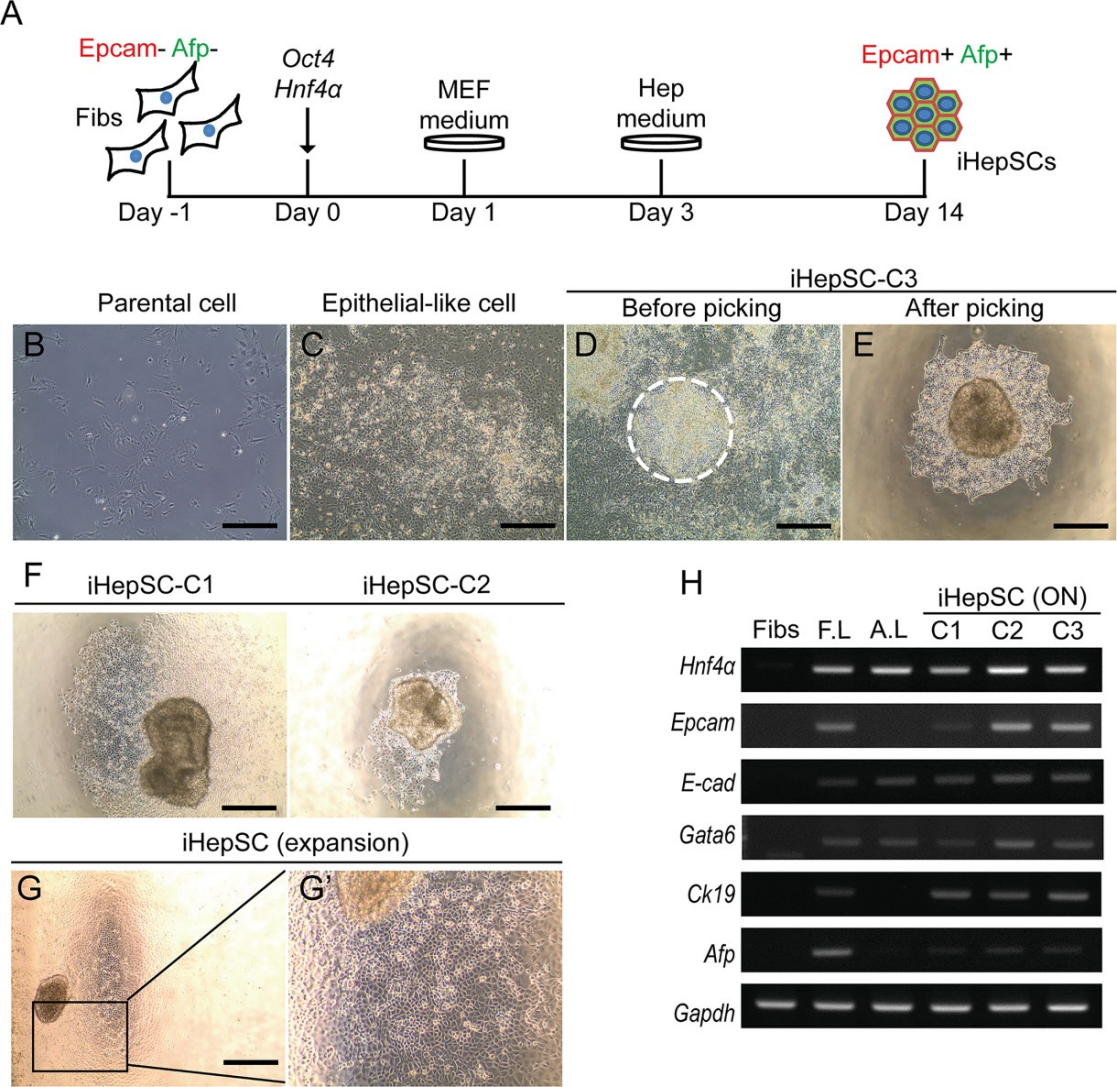
Generation of induced hepatic stem cells (iHepSCs) from fibroblasts by overexpressing of *Oct4* and *Hnf4α*. (A) Direct conversion scheme for iHepSCs. Mouse fibroblasts (Fibs) were infected with lentivirus expressing *Oct4* and *Hnf4α* (2F). The virus contained medium was changed to fresh MEF medium at one day post-infection (dpi). The culture medium was switched to HEP medium (at day 3), and maintained until iHepSC colonies appeared. (B) Image of parental cells before viral infection. (C) Image of typical epithelial cell clusters at 10 dpi. (D) Image of iHepSC colonies at day14. (E) Image of iHepSC-C3 after picking. The white dashed circle in (D) indicates the isolated colony. Scale bars: 250 μm. (F) Images of iHepSC-C1 and iHepSC-C2. Scale bars: 250 μm. (G-G’) Expansion of isolated iHepSC colony. The black box enlarged in (G’). Scale bars: 250 μm. (H) Gene expression analysis of HepSC and hepatocyte- specific markers in fibroblasts (Fibs), three iHepSC clones (C1~3) and liver tissues (E14.5 fetal liver: F.L; adult liver: A.L).

*Oct4* and *Hnf4α*–induced hepatic stem cells (iHepSCs) from fibroblasts were generated by a series of steps (Fig 1A). We confirmed that parental cells were not contaminated with hepatocytes or hepatic stem cells by immunostaining with hepatic lineage markers (S1A Fig). Ten days after transduction of *Oct4* and *Hnf4α* (2F) to the fibroblast, we observed typical epithelial-like cell morphology in 2F-transduced cells, which are distinct from the parental cells (Fig 1B and 1C). Then at day 14 2F-transduced epithelial-like cells formed colony-like structures (Fig 1D, dashed circle). However, we observed no morphological changes when either *Oct4* or *Hnf4α* was used alone (S1B Fig). The colonies were mechanically isolated and transferred to the collagen-coated dish (Fig 1E). Finally, we established three iHepSC clones and named them iHepSC-C1, iHepSC-C2, and iHepSC-C3 (Fig 1F and 1E). iHepSCs could maintain homogeneously on the collagen-coated dish (Fig 1G). iHepSCs showed a cuboidal morphology with clear nuclei and a high nucleus/cytoplasm ratio. iHepSC clones (C1-C3) commonly expressed hepatoblasts and early hepatocyte-specific markers, including *Hnf4α*, *Epcam*, *E-cad*, *Gata6*, *Ck19*, and *Afp* (Fig 1H). We also confirmed the integration of both *Oct4* and *Hnf4α* transgenes within these isolated iHepSC clones by genotyping PCR (S1D Fig). We evaluated the population-doubling times of iHepSC clones during in vitro expansion (n = 3). The population doubling time of iHepSC-C3 was 29.19 ± 0.17 hours, which is significantly less than other clones (38.44 ± 0.28 hours for iHepSC-C1; 38.25 ± 0.25 hours for iHepSC-C2) and parental cells (83.56 ± 3.03 hours), respectively. Further analysis of iHepSC was performed in detail with iHepSC-C3 among the iHepSC clones (S1E Fig). These data indicate that *Oct4* and *Hnf4α* are sufficient to convert somatic cells into HepSCs.

### Characterization of iHepSCs

**Fig 2:**
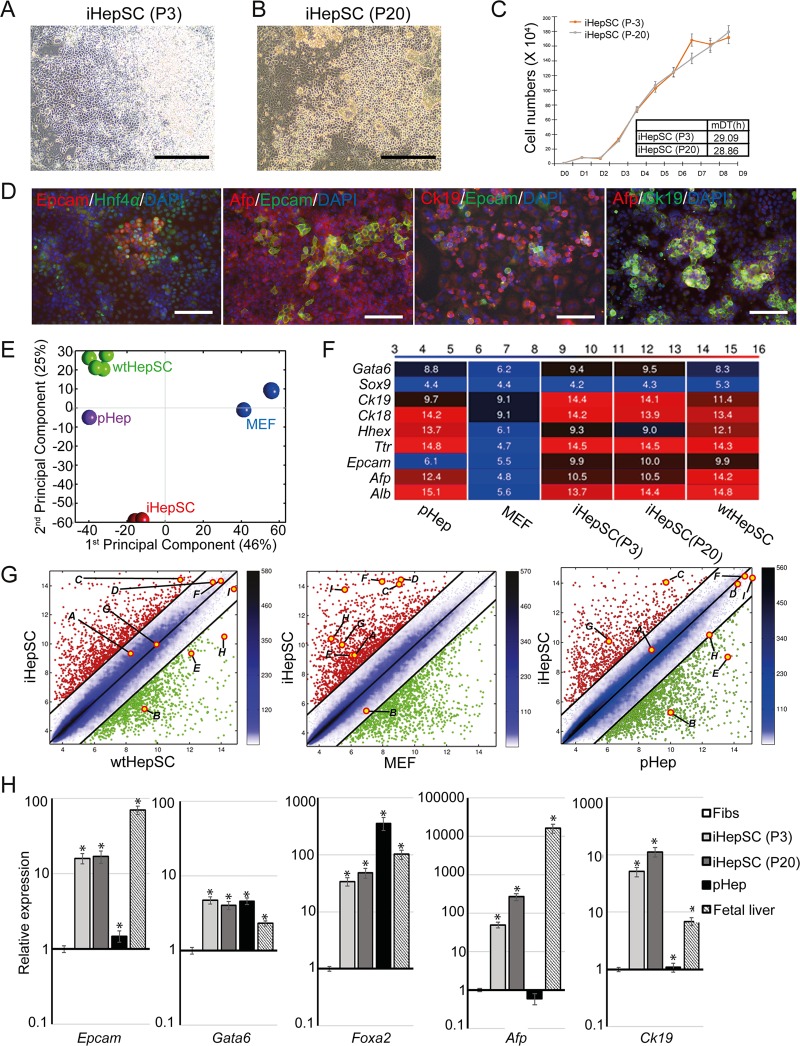
Characterization of iHepSCs. (A-B) Images of iHepSCs at early passage (passage 3, P3, A) and late passage (passage 20, P20, B). Scale bar: 250 μm. (C) Growth curves for iHepSCs at early and late passage. The points refer to the cell numbers of iHepSCs on each day. The table represents the mean doubling time (mDT) of early (P3) and late (P20) iHepSCs for ten days. The experiment was carried out triplicate (n = 3). (D) Immunofluorescence images of iHepSCs stained with HepSC markers (Epithelial cell adhesion molecule: Epcam; hepatocyte nuclear factor 4 α: Hnf4; α-fetoprotein: Afp; Cytokeratin19: Ck19). The cells were counterstained with DAPI. Scale bar: 150 μm. (E) PCA analysis of mouse embryonic fibroblasts (MEF), wild-type HepSC (wtHepSC), primary hepatocytes (pHep) and iHepSCs (early and late passage). (F) Heatmap analysis of the selected genes involved in the hepatic lineage. The color bar in the top codifies the gene expression level in log2 scale. (G) Pairwise scatter plots of samples; iHepSC vs. wtHepSC, iHepSC vs. MEF, and iHepSC vs. pHep. Hepatic markers were labelled as follow; (a) *Gata6*, (b) *Sox9*, (c) *Ck19*, (d) *Ck18*, (e) *Hhex*, (f) *Ttr*, (g) *Epcam*, (h) *Afp*, (i) *Alb*. (H) Expressional levels of hepatic genes of the parental cell (Fibs), fetal liver, iHepSC (early and late passage) and pHep. The transcriptional levels were normalized to the housekeeping gene (*Gapdh)*. Error bars indicate standard errors from triplicate samples (n = 3). *, P<0.05.

To investigate whether iHepSCs possess the characteristics of hepatic stem cell, we analyzed iHepSCs in detail. iHepSCs in both early (P3) and late (P20) passage showed a similar morphology (Fig 2A and 2B) and had a similar doubling time (DT) (Fig 2C). The mean of DT (n = 3) was 29.09 ± 0.63 and 28.86 ± 0.17 hours, respectively. Therefore, the results showed that iHepSCs could maintain the self-renewal capacity in long-term culture. Immunostaining assay revealed that iHepSCs maintain the protein expression of hepatic stem cell markers including Epcam (31.2 ± 10.3%), Hnf4α (81.3 ± 5.0%), Afp (83.3 ± 2.7%), and Ck19 (93.2 ± 0.8%) (Fig 2D, S8, S12, and S13 Figs). The exogenous expression of *Oct4* and *Hnf4α* mRNA was silenced in both early and late passages (S1G Fig). To determine whether parental or pluripotent cells are present in the iHepSCs population, we immunostained iHepSCs with fibroblast (Vimentin) or pluripotency marker (Oct4). We could not observe Vimentin-positive or Oct4-positive cells in the iHepSCs population (S1C and S1F Fig). To evaluate the overall molecular status of iHepSCs, we analyzed the global gene expression profiles of iHepSCs (early and late passages) compared with freshly isolated primary hepatocytes (pHep) and the wild-type hepatic stem cells (wtHepSCs) that have been reported in a previous study [28]. Principal Component Analysis (PCA) and heatmap analysis of wtHepSC, pHep and iHepSC showed similar global transcriptome profiles, whereas parental cell (MEF) had a distinct gene expression pattern (Fig 2E and S2A Fig). Moreover, the distinct heatmap and the pairwise scatter plots showed high similarity between wtHepSC and iHepSC in hepatic lineage-specific genes, including *Gata6*, *Sox9*, *ck19*, *ck18*, *Hhex*, *Ttr*, *Epcam*, *Afp*, and *Alb* (Fig 2F–2G and S2B Fig). We analyzed the gene ontology (GO) term enrichment profiling of iHepSCs using a set of genes that are involved in various hepatic functions, such as epithelial cell differentiation, lipid biosynthetic process, glucose-, xenobiotic-, bile acid-, steroid-, fatty acid- and cholesterol metabolic process. Numerous hepatic functional genes were highly enriched in iHepSCs compared to MEF (S2C Fig and S1 Table). We also performed a gene set enrichment analysis between iHepSC and wtHepSC. We found that iHepSC was abundant in genes that are related to inflammatory response, xenobiotic stimulus regulation, and digestion compared to wtHepSCs (S2D Fig). To validate the microarray data, we examined the transcriptional levels of hepatic lineage-specific genes including, *Epcam*, *Gata6*, *Foxa2*, *Hnf4a*, and *ck19* by qPCR (n = 3). Following the microarray data, the mRNA expressions of hepatic lineage-specific genes were up-regulated in both early and late iHepSCs relative to the fibroblasts (Fig 2H). These results thus demonstrate that iHepSCs are expandable and sustain HepSC phenotypes in long-term culture.

### Differentiation of bipotential iHepSCs into hepatocyte- and cholangiocyte-like cells

**Fig 3:**
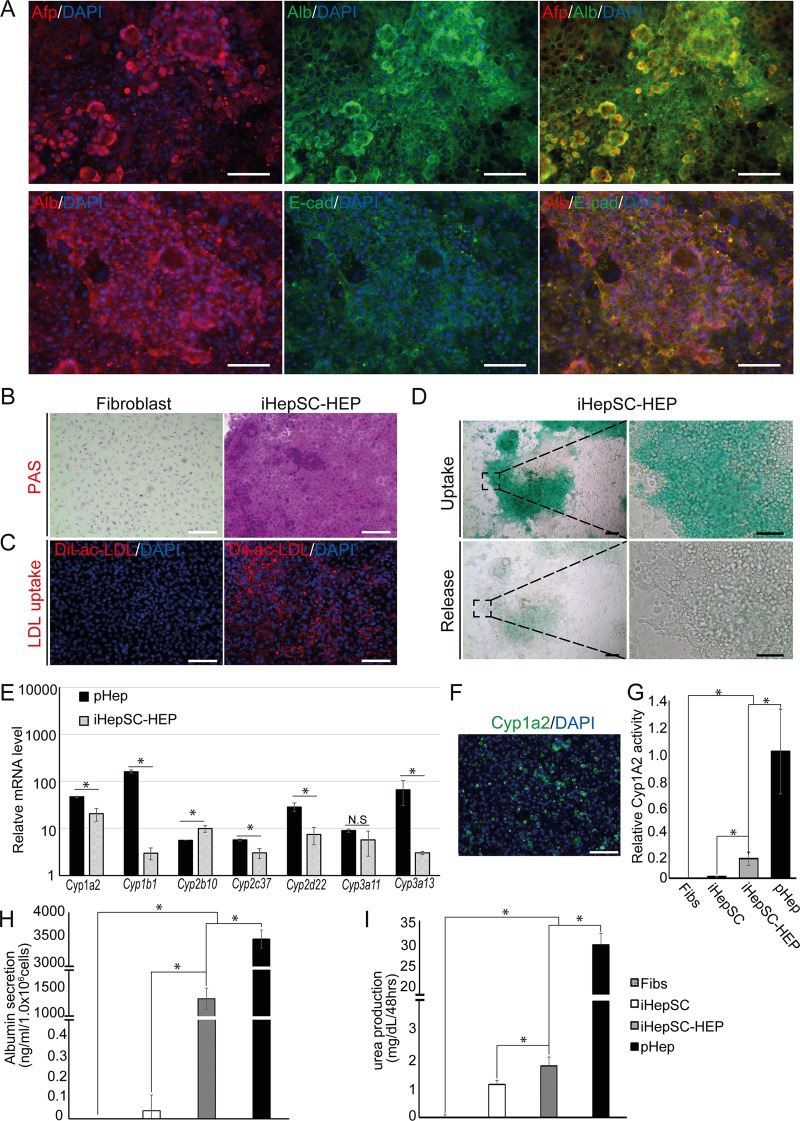
Characterization of iHepSC derived-hepatocyte (iHepSC-HEP). (A) Immunofluorescence images of iHepSC-HEP expressing hepatocyte markers (α-fetoprotein: Afp; Albumin: Alb; E-cadherin: E-cad). The nucleus was stained with DAPI. Scale bar: 150 μm. (B) Image of Periodic acid-Schiff (PAS) stained iHepSC-HEP. (C) Image of acetylated low-density lipoprotein (LDL) uptake in iHepSC-HEP. (D) Detoxification capacity evaluated by indocyanine green (ICG) uptake and release assay. The nucleus was stained with Hematoxylin and DAPI. Fibroblasts were used as negative control. The black dashed boxes were enlarged. Scale bars: 150 μm. (E) The mRNA expression levels of Cytochrome P450 (CYP) family in iHepSC-HEP and primary hepatocyte (pHep) were analyzed by qPCR. iHepSC-HEP and pHep were incubated with inducers (3-methylcholanthrene and Rifampicin) for 72 hrs. The mRNA expression levels were normalized by the levels in cells without inducer treatment. Error bars indicated standard errors from triplicate samples (n = 3). *, P<0.05. (F) Immunostaining image of iHepSC-HEP stained with Cyp1a2. The nucleus was stained with DAPI. Scale bar: 150 μm. (G) The activity of Cyp1a2 in response to 3-Methylcholanthrene (50 μM). (H-I) The amount of Albumin secretion (H) and Urea production (I) of iHepSC-HEP in comparison with fibroblast (Fibs), iHepSC and pHep were examined by ELISA. Error Bars indicate standard errors from triplicate samples (n = 3). *, P<0.05.

We investigated whether the expandable iHepSCs possess the bipotency to differentiate into hepatocytes and cholangiocytes. To differentiate iHepSC into hepatocytes, we plated iHepSCs on a collagen-coated dish in the HEP medium as described previously [11, 13]. We evaluated the expression of mature hepatocyte markers in hepatocyte-like cells derived from iHepSC (iHepSC-HEPs) (S3 Fig). Immunostaining revealed that iHepSC-HEPs expressed hepatic markers including Afp (64.8 ± 4.4%), Albumin (93.8 ± 3.2%), and E-cadherin (84.1 ± 1.8%) (Fig 3A and S9 Fig), whereas Epcam (hepatic stem cell marker) and Ck19 (cholangiocyte marker) were not detected (S4 Fig). Also, we confirmed the glycogen storage properties of iHepSC-HEPs by Periodic acid–Schiff (PAS) staining (Fig 3B). iHepSC-HEPs showed uptake of DiI-labelled acetylated low-density lipoprotein (DiI-ac-LDL) into the intracellular region via LDL receptor-mediated endocytosis (Fig 3C). To evaluate the detoxification function of iHepSC-HEPs, we conducted the indocyanine green (ICG) assay as described previously [10–14]. The absorbed ICG (green) in iHepSC-HEPs is significantly removed after six hours in ICG-free culture medium (Fig 3D). Expression levels of Cytochrome P450 (CYP) family members including *Cyp1a2*, *Cyp2b10*, *Cyp2c37*, *Cyp2d22*, *Cyp2e1*, *Cyp3a11*, *Cyp3a13* and *Cyp7a1* in iHepSC-HEPs were compared with primary hepatocytes (pHep) (S5A Fig). To investigate CYP gene induction, we cultured iHepSC-HEPs and pHep with CYP inducers, 3-methylcholanthrene and Rifampicin, and analyzed mRNA expression of CYP family genes in iHepSC-HEPs and pHep (n = 3). CYP family genes, such as *Cyp1a2*, Cyp1b1, *Cyp2b10*, *Cyp2c37*, *Cyp2d22*, *Cyp3a11*, and *Cyp3a13*, were significantly up-regulated in iHepSC-HEPs after chemical induction (Fig 3E). We could observe Cyp1a2 protein expression in iHepSC-HEPs (Fig 3F). In addition, Cyp1a2 activity (n = 3) was significantly increased after hepatic maturation (Fig 3G). The levels of albumin secretion (n = 3) and the mRNA expression of *albumin* (n = 3) were significantly increased in iHepSC-HEPs compared to iHepSCs (Fig 3H and S5B Fig). iHepSC-HEPs showed significantly higher urea production (n = 3) and expression of urea-cycle related genes such as *Arg1*, *Otc*, *Asl*, *Ass1*, and *Cps1* (n = 3) (Fig 3I and S5C Fig). Therefore, iHepSC-HEPs possess the hallmarks of hepatic functionality including glycogen storage, LDL uptake, ICG clearance, CYP activity induction, albumin secretion, and urea production. Overall, these data indicate that iHepSCs can be differentiated into functional hepatocytes.

**Fig 4:**
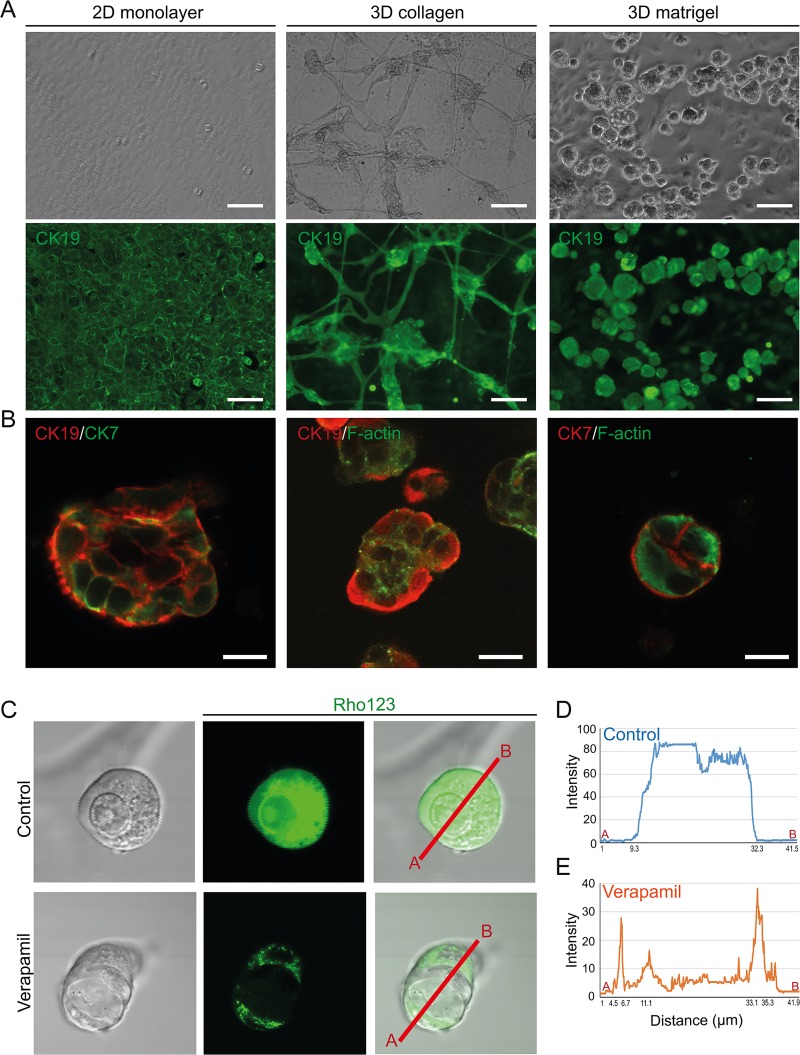
Characterization of iHepSC derived-cholangiocyte-like cells (iHepSC-CLCs). (A) Phase- contrast images of iHepSC-CLCs in different culture conditions (upper panel). Immunofluorescence images of iHepSC-CLCs stained with antibody against Cytokeratin19 (Ck19) (lower panel). Images of iHepSC-CLCs on 2D culture (left column). The tubule-like structure of iHepSC-CLCs cultured in the 3D collagen gel (middle column). The Cyst-like structure of iHepSC-CLCs cultured in the 3D Matrigel (right column). Scale bar: 150 μm. (B) Immunofluorescence analysis of iHepSC-CLCs co-stained with cholangiocyte specific markers, Ck19 and Cytokeratin7 (Ck7). Localization of F-actin (phalloidin) in the apical region of iHepSC-CLCs. The cells were counterstained with DAPI. Scale bar: 25 μm. (C) Transportation of rhodamine 123 (Rho 123) into the central lumen of the cyst (upper panel). The Mdr1 inhibitor, verapamil (10 μM), blocks Rho123 transportation (Lower panel). (D-E) Quantifications of the fluorescence intensity of Rho 123 in the cysts. The fluorescence intensity values along the A-B axis shown in (C) were measured using Olympus software and analyzed by image J.

To differentiate iHepSC into cholangiocytes, we optimized the cholangiocyte differentiation method in three-dimensional (3D) culture system using collagen or Matrigel with cholangiocyte culture medium as described previously [27]. Cholangiocyte-like cells derived from iHepSC (iHepSC-CLCs) cultured on collagen gel and Matrigel formed branching, tubular and round cystic structures and these structures uniformly expressed cholangiocyte specific-marker (CK19) (Fig 4A). However, iHepSCs cultured in a 2D monolayer culture system were insufficient to develop the tubular or cystic structures in Ck19- positive cholangiocytes. Additionally, we evaluated apical-basal polarity in the cystic iHepSC-CLCs by confocal microscopy. F-actin bundles were detected in the inner lumen of the cystic iHepSC-CLCs, which expressed cholangiocyte markers (CK19 and CK7) (Fig 4B). qPCR revealed that the transcriptional levels of cholangiocyte-specific markers including *Sox9*, *Ck19*, *Ck7*, *Hnf1β*, *Hnf6*, *Onecut2*, *Slc4a2*, *Slc10a2*, and *Aqp1* (n = 3) were upregulated in iHepSC-CLCs compared to iHepSCs (S7 Fig). Rhodamine 123 has been used to measure the activity of multidrug resistance protein 1 (Mdr1), a cholangiocyte surface glycoprotein as previously described [36]. We incubated iHepSC with rhodamine 123 to evaluate the secretory function of iHepSC-CLCs and observed transported rhodamine 123 in the lumen of the cystic iHepSC-CLCs (Fig 4C). Meanwhile, in the presence of Verapamil, an Mdr1 inhibitor, the Mdr1 transport potential of cystic iHepSC-CLCs was blocked (Fig 4D and 4E) thereby showing the presence of functional Mdr1 transporter in the iHepSC-CLCs. The apical-basal polarity of iHepSC-CLCs is associated with cyst development that can efflux Mdr1 substrates from the basal to the apical luminal space. These results demonstrate that iHepSCs can differentiate into cholangiocyte-like cells that have typical apical-basal polarity and secretory function.

### Therapeutic effects of iHepSC on carbon tetrachloride (CCl_4_)-induced liver fibrosis

**Fig 5:**
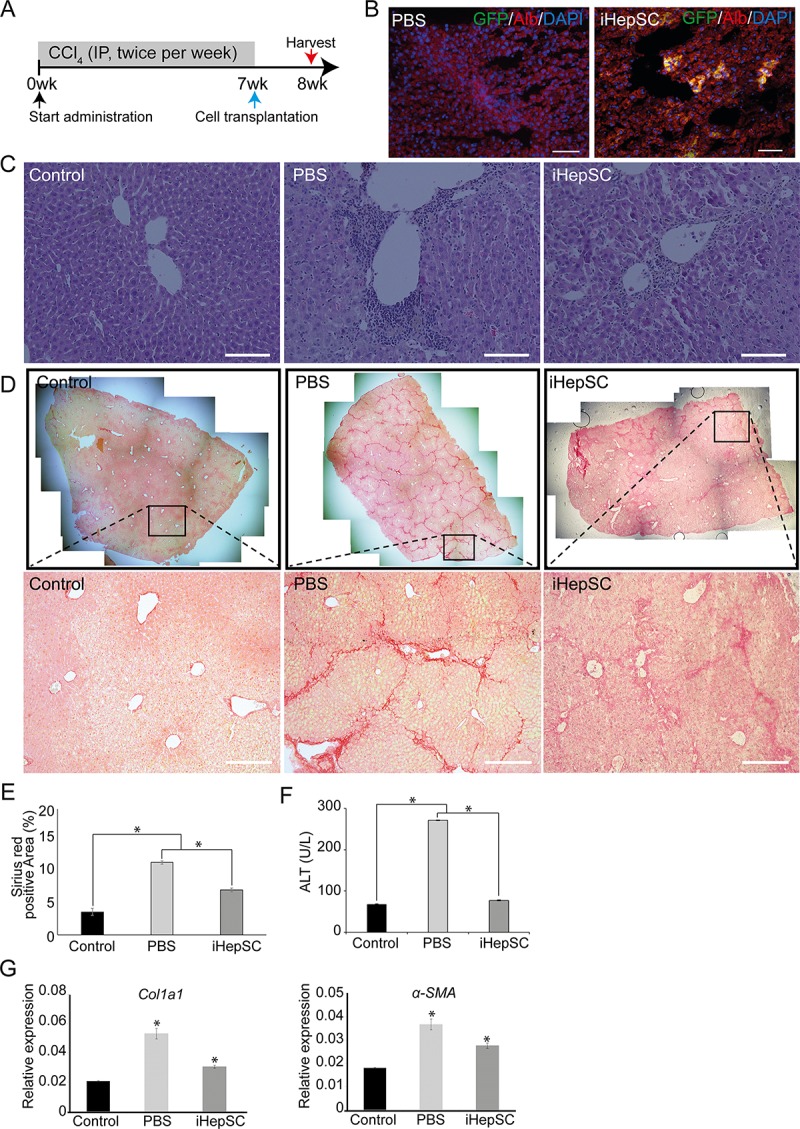
Therapeutic effects of transplanted iHepSCs in CCl_4_-induced chronic liver fibrosis model. (A) Scheme for *in vivo* experiment. (B) Immunofluorescence images of GFP-labeled iHepSCs in liver sections of the mice. The yellow area represents the successful engraftment of iHepSCs into the liver through the spleen (4.07 ± 0.33%). The nucleus was stained with DAPI. Scale bar: 150 μm. (C-D) Images of H&E and Sirius red staining for liver sections of fibrosis mice model. The black box enlarged in below. Scale bar: 150 μm. (E) Quantification of the fibrotic area (%) of liver isolated from (D). Sirius red staining shows collagen deposition of liver. Fibrous positive area (%) was analyzed by using software image J 1.51J. *, P<0.05. (F) Serum levels of alanine aminotransferase (ALT) (n = 9). *, P<0.05. (G) Gene expression analysis of fibrotic genes (*Alpha-1 type 1 collagen*: *Col1a1; alpha-smooth muscle actin*: *α-SMA*) in the liver tissues by qPCR. The transcriptional levels were normalized by the housekeeping gene (*Gapdh*). Error bars indicated standard errors from triplicate samples (n = 3). *, P<0.05.

To examine whether iHepSCs have therapeutic effects on liver disease, we transplanted iHepSCs into mice with CCl_4_-induced liver fibrosis by intra-splenic injection (Fig 5A). Administration of CCl_4_ is a widely used model to study acute/chronic liver injury [2, 33, 37, 38]. Seven weeks after repeated exposure of CCl_4_, the green fluorescent protein (GFP)-labeled iHepSCs were transplanted into the liver through the spleen. We observed albumin and GFP double-positive iHepSCs in liver tissue (Fig 5B and S10 Fig). Liver fibrosis was evaluated histologically using Hematoxylin and eosin (H & E)-staining after cell transplantation. In contrast to PBS-injected mice, iHepSC-injected mice showed a decreased number of infiltrating T lymphocytes (Fig 5C). To evaluate the protective effects of iHepSCs in liver fibrosis, we conducted Sirius red staining to analyze collagen accumulation. In comparison to normal mice, increased accumulation of collagen fibers was observed throughout the whole liver section of PBS-injected mice (Fig 5D). We observed pseudo-lobule like structures formed by collagen fibers in the liver tissue of PBS-injected mice. Quantitative image analysis of collagen fibers in liver tissues showed that the percentage of fibrotic areas significantly decreased after iHepSC transplantation (7.2 ± 0.3%) in comparison to PBS-injected mice (11.5 ± 0.2%) (Fig 5E). The histological analysis demonstrates that the liver in iHepSC-injected mice had a reduction in infiltration of lymphocytes and the accumulation of collagen fibers compared to PBS-injected mice. Serum levels of alanine aminotransferase (ALT) of iHepSC-injected mice were similar to those of normal mice (Fig 5F). qPCR analysis showed changes in the expressional levels of fibrosis and inflammatory markers in the liver of CCl_4_-injected mice. The transcription levels of genes involved in fibrosis, ECM modulation, and inflammatory response in iHepSC-transplanted mice livers were significantly lower than those in the liver of PBS-injected mice (Fig 5G and S11 Fig). To evaluate the tumorigenicity of iHepSCs, we subcutaneously injected iHepSCs into athymic nude mice (n = 5). We could detect no tumors in any recipient mice (S6 Fig). These results suggest that iHepSCs have therapeutic potential for chronic liver damage in CCl_4_-induced liver fibrosis mice.

## Discussion

In this study, we demonstrate that ectopic expression of *Oct4* and *Hnf4α* is sufficient to induce cell fate conversion of mouse fibroblasts into hepatic stem cells (iHepSCs). iHepSCs express hepatic stem cell-specific markers and possess self-renewal and bipotential properties to differentiate into hepatocytes (iHepSC-HEPs) and cholangiocyte-like cells (iHepSC-CLCs). Huang and colleagues have demonstrated that increased the numbers of epithelial colonies by removing *Hnf4α* from the combination of transcription factors for hepatocyte induction [11]. Although *Hnf4α* may inhibit cell proliferation [39], it is highly expressed at the hepatoblast stage of differentiating PSC [40]. Previous studies have demonstrated that *Hnf4α* is essential for liver development by controlling the expression of hepatic specific genes such as *Hnf1α/β*, *Hnf6*, and *Gata6*, which are involved in glucose, fatty acid, cholesterol, bile acid metabolism, and drug metabolism [41, 42]. Genetic modification experiments revealed that *Hnf4α* is essential for human hepatic specification [41] and mouse fetal liver development [42].

*Oct4* is known to be the key transcription factor in maintaining pluripotency. Zhao and colleagues demonstrated that *Oct4*, a transcription factor required to maintain pluripotency, reduce the risk of apoptosis by inhibiting cyclin-dependent kinase 1 activity that leads to premature mitotic entry in PSC [22]. We believe that *Oct4* may play a crucial role in maintaining rapid cell proliferation in the iHepSCs. Besides its crucial role in maintaining pluripotency, *Oct4* has a diversified function in different circumstances. Aksoy and colleague reported that *Oct4* is involved in endoderm lineage commitment by partnering with *Sox17* and increases the expression of primitive endoderm related genes, such as *Gata4* and *Gata6*, that are associated with endoderm development in mice [43]. Previous studies have shown that the combination of *Oct4* and lineage-specific culture conditions is sufficient to generate induced neuronal and blood progenitor cells through direct conversion [44, 45]. We previously demonstrated that *Oct4* is sufficient to convert somatic cell to oligodendrocyte progenitor cells [23]. In this study, we found that the combination of *Oct4* and *Hnf4α* with appropriate culture conditions is sufficient to induce hepatic cell fate commitment and maintain clonally expandable cell line in the transdifferentiation process.

Several groups have reported that transplantation of hepatocytes differentiated from pluripotent stem cells (PSCs) [6, 7] or that are directly converted from fibroblasts [15, 38] improved liver function in acute liver injury. However, the limited proliferation of these cells has been the major problem for acquiring enough cell number that can be used in cell-based therapy [17–20]. Meanwhile, several researchers have reported that expandable induced hepatocyte-like cells (iHeps) can be generated by overexpression of *Myc* and silencing of *p53* [10], overexpression of SV40 large T antigen [12] or knockdown of *p19*^*arf*^ [11]. HepSCs are self-renewing bipotent stem cells, which contribute to liver development and liver regeneration after injury [17–20]. Therefore, HepSCs can be an alternative cell source for cell-based therapy.

Several studies reported that either endogenous hepatocytes or liver resident HepSCs could contribute to liver regeneration in liver injury models [46–50]. We observed the transplanted iHepSCs co-localized with albumin in liver sections (Fig 5B). This data suggests that these iHepSCs rapidly differentiated into hepatocytes that recover liver function. Further studies may explore the mechanism of cell fate determination of engrafted iHepSC and the therapeutic effects in CLD. The results demonstrate that the transplanted iHepSCs could recover liver damage in liver fibrosis model by reducing histological grading of fibrosis in the liver and the serum ALT levels in iHepSC-transplanted mice (Fig 5C–5F). Furthermore, we demonstrate that iHepSCs have anti-fibrotic and anti-inflammatory effects in CCl_4_-induced CLD by reducing the transcriptional level of genes involved in fibrosis, ECM modulation and inflammatory response in contrast to the livers of PBS-injected mice (Fig 5G and S11 Fig). However, understanding of the mechanism of anti-fibrotic and anti-inflammatory induced by iHepSC in liver fibrosis should be elucidated in further studies.

In conclusion, we demonstrated that *Oct4*, *Hnf4α*, and defined culture conditions are sufficient to directly convert somatic cells to self-renewal and bipotent iHepSCs that can differentiate into functional hepatocytes and cholangiocytes. Importantly, iHepSCs showed the therapeutic effects in a CCl_4_ induced liver fibrosis model. Further studies are necessary to study the therapeutic effects of iHepSCs in different chronic liver disease models by using precise lineage tracing transgenic mice. Our study opens the perspective to generate human iHepSCs that may be applied for regenerative cell-based therapies and cell-based screening platforms for the treatment of liver diseases.

## Supporting information

S1 Fig Selection of iHepSC clone in the process of cell fate conversion. (A) Immunostaining analysis of mouse fibroblasts (Parental cells) with hepatic markers (E-cadherin: E-cad; α-fetoprotein: Afp; albumin: Alb; cytokeratin19: CK19). The nucleus was stained with DAPI. Scale bars: 150 μm. (B) Cell morphologies of TF induced fibroblasts (Fibs) at 21 days after transduction. Fibroblasts underwent proliferation arrest and cell death three weeks after transduction. Images indicated morphologies of no induction (none), mock-infection (GFP), single factor induction groups (*Oct4* and *Hnf4α* only). Scale bars: 250 μm. (C) Immunostaining analysis of mouse fibroblasts and iHepSCs with hepatic marker (Alb) and fibroblast marker (Vimentin: Vim). The nucleus was stained with DAPI. Scale bars: 150 μm. (D) Genotyping of picked iHepSCs. Three iHepSC clones were mechanically isolated from infected plates. Genomic PCR analysis shows *Oct4* and *Hnf4α* transgene insertion in the genome of three iHepSC clones (#1, #2, #3). Parental cells were used as negative controls. (E) Population doubling times of iHepSC clones (#1, #2 and #3) and parental cells (Fib). The values of the bar represent the mean of doubling time (mDT) of iHepSC clones and parental cells during 10 days. The experiment was carried out triplicate. *, P<0.05. N.S, not significance. (F) Immunostaining analysis of iHepSCs and mouse embryonic stem cells derived from OG2-ROSA transgenic mouse with pluripotency marker (Oct4) and hepatic marker (Alb). The cells were counterstained with DAPI. Scale bars: 150 μm. (G) Silencing of the exogenous *Oct4* and *Hnf4α* genes in 2F iHepSCs. The expression levels were determined by qPCR using primer specific for transgenic transcripts. Transgenic expression levels of fibroblasts were compared with those in 5 days post-infection and on iHepSC clones at passage 3 and 20. Transcript levels were normalized to *Gapdh*. Error bars indicated standard errors from triplicate samples (n = 3). *, P<0.05.
(TIF)

S2 Fig Global gene expression profiles of *Oct4* and *Hnf4α* induced HepSCs. (A) Heatmap analysis of the global gene expression profiles of fibroblast (MEF), freshly isolated hepatocyte (pHep), iHepSC (P3), iHepSC (P20), and wild type HepSC (wtHepSC 1–4). The color bar in the top codifies the gene expression in log2 scale. (B) Pairwise scatter plots of samples; pHep vs wtHepSC (upper), pHep vs wtHepSC (middle), and wtHepSC vs MEF (lower). Hepatic markers were labelled as follow; (a) *Gata6*, (b) *Sox9*, (c) *Ck19*, (d) *Ck18*, (e) *Hhex*, (f) *Ttr*, (g) *Epcam*, (h) *Afp*, (i) *Alb*. (C) Functional enrichment analysis of parental cell and iHepSCs. On the left side of the panel, Plot bar of the -log10(p) of the significantly enriched terms of MEF-<-iHepSC-Log2(16). There are 366 differentially Down-regulated transcripts. On the right side of the panel, Plot bar of the -log10(p) of the significantly enriched terms of MEF->-iHepSC-Log2(16). There are 467 differentially Up-regulated transcripts. The longer the bar, the higher is the statistical significance of the enrichment. The p-values are written in parenthesis. (D) Functional enrichment analysis of wtHepSC and iHepSC. On the left side of the panel, Plot bar of the -log10(p) of the significantly enriched terms of wtHepSC-<-iHepSC-Log2(16). There are 239 differentially Down-regulated transcripts. On the right side of the panel, Plot bar of the -log10(p) of the significantly enriched terms of wtHepSC->-iHepSC-Log2(16). There are 218 differentially Up-regulated transcripts. The longer the bar, the higher is the statistical significance of the enrichment. The p-values are written in parenthesis.
(TIF)

S3 Fig Gene analysis against hepatocyte markers of iHepSC-HEPs, primary hepatocytes (pHeps), and parental cells (Fib). Gene analysis against hepatic stem cell and hepatocyte-specific markers, such as endogenous *Hnf4α*, *Foxa2*, *E-cadherin (Ecad)*, *Ck8*, *Ck18*, *Alb*, *transthyretin (Ttr)*, *tyrosine aminotransferase (Tat)*, *Alpha-1 antitrypsin (Aat)* and *glucose 6-phosphatase (G6Pase)* of iHepSC-HEPs, pHeps, and Fibs. Fibs and pHeps were used as negative and positive controls.
(TIF)

S4 Fig The differentiated iHepSC-HEPs have precisely analyzed their hepatic phenotype. Immunostaining analysis revealed that iHepSC-HEPs negatively stained with hepatic stem cell markers (Epithelial cell adhesion molecule: Epcam) and cholangiocyte marker (Cytokeratin19: Ck19). The nucleus was stained with DAPI. Scale bar: 150 μm.
(TIF)

S5 Fig Quantitative PCR analyses of Cytochrome P450 (CYP) family, albumin, and urea cycle pathway in iHepSC-HEPs. (A) Gene expression analysis against CYP family, such as *Cyp1a2*, *Cyp2b10*, *Cyp2c37*, *Cyp2d22*, *Cyp2e1*, *Cyp3a11*, *Cyp3a13* and *Cyp7a1* in iHepSC-HEP (black) and pHep (white) relative to parental cells. The transcriptional levels were normalized to a housekeeping gene (*Gapdh*) and represented in the logarithmic scale. Error bars indicated standard errors from triplicate samples (n = 3). (B-C) Gene expression analysis of *Albumin* (B) and urea cycle pathway (C) in iHepSC, iHepSC-HEP, and pHep relative to parental cells (fibs). The transcriptional levels were normalized to the housekeeping gene (*Gapdh*) and represented in the logarithmic scale. Error bars indicated standard errors from triplicate samples (n = 3).
(TIF)

S6 Fig Evaluation of tumorigenicity in iHepSC transplanted mice. iHepSCs (2 x 10^6^ cells/mouse) were injected subcutaneously of the dorsal flank of athymic nude mice (n = 5). At 12 weeks after the subcutaneous injection, mice were sacrificed for analysis. No tumors were detected in recipient mice.
(TIF)

S7 Fig Gene analysis against cholangiocyte-specific markers of iHepSC, bile duct, and cholangiocyte derived from iHepSC (iHepSC-CC). Gene expression analysis against cholangiocyte-specific markers including *Sox9*, *Ck19*, *Ck7*, *Hnf1β*, *Hnf6*, *Onecut2*, *Slc4a2*, *Slc10a2*, and *Aqp1* in iHepSC-CC, iHepSC, and bile duct by qPCR. Mouse bile duct isolated from C57BL/6J mouse used as positive controls. The transcriptional levels were normalized to the housekeeping gene (*Gapdh*). Error bars indicated standard errors from triplicate samples (n = 3). *, P<0.05.
(TIF)

S8 Fig Multi-channel images of iHepSC stained with hepatic stem cell (HepSC) markers. Multi-channel images of iHepSC stained with HepSC markers including Epithelial cell adhesion molecule: Epcam; hepatocyte nuclear factor 4 α: Hnf4; α-fetoprotein: Afp; Cytokeratin19: Ck19. The nucleus was stained with DAPI. Scale bar: 150 μm.
(TIF)

S9 Fig Multi-channel images of iHepSC-HEP stained with hepatic markers. Multi-channel images of iHepSC-HEP stained with α-fetoprotein: Afp; Albumin: Alb; E-cadherin: E-cad. The nucleus was stained with DAPI. Scale bar: 150 μm.
(TIF)

S10 Fig Multi-channel images of iHepSCs stained with albumin and GFP in liver sections. Multi-channel images of iHepSCs stained with anti-GFP (Green) and anti-albumin (Red) at liver sections of both PBS injected mine (A) and iHepSCs transplanted mice (B). The nucleus was stained with DAPI. Scale bar: 150 μm.
(TIF)

S11 Fig Transplanted iHepSC reduce ECM regulation and inflammatory response in liver fibrosis. (A) The transcription levels of genes related to ECM modulation including, *Tissue inhibitor of metalloprotease protein1*: *Timp1; Matrix metalloproteinases2*: *Mmp2; Matrix metalloproteinase9*: *Mmp9* in liver tissues by qPCR. The transcriptional levels were normalized by the housekeeping gene (*Gapdh*). Error bars indicated standard errors from triplicate samples (n = 3). *, P<0.05. (B) The transcription levels of genes involved in an inflammatory response including, *Tumor necrosis factor-alpha*: *Tnfα; Interleukin6*: *Il6; Interleukin1 beta*: *Il1β* in liver tissues by qPCR. The transcriptional levels were normalized by the housekeeping gene (*Gapdh*). Error bars indicated standard errors from triplicate samples (n = 3). *, P<0.05.
(TIF)

S12 Fig Immunostaining images of primary hepatocytes with hepatic markers. Immunostaining images of primary hepatocytes stained with hepatic markers including, Afp, Alb, E-cad, Hnf4α, Ck19, and Cyp1a2. The nucleus was stained with DAPI. Scale bar: 150 μm.
(TIF)

S13 Fig Immunostaining images of primary hepatocytes and iHepSC-HEP with hepatic markers and Isotype controls. Immunostaining images of primary hepatocytes and iHepSC-HEP stained with hepatic markers including, Hnf4α, E-cad, and alb. The nucleus was stained with DAPI. Mouse IgG, Goat IgG, Rat IgG2a, Rabbit IgG, and Mouse IgG antibodies were used as isotype controls. Scale bar: 150 μm.
(TIF)

S1 Table Differentially expressed gene analysis among liver metabolic activity of iHepSC VS. Parental cells (MEF) and primary hepatocyte (pHep) VS. MEF. (DOCX)

S2 Table Lists of primers sequences. (DOCX)

S3 Table List of primary antibodies. (DOCX)
