# Supplementary figures and images for: Oct4 and Hnf4α-induced hepatic stem cells ameliorate chronic liver injury in liver fibrosis model
Source: PLoS One. 2019 Aug 12;14(8):e0221085. doi: 10.1371/journal.pone.0221085 (PMC6690533; doi:10.1371/journal.pone.0221085)

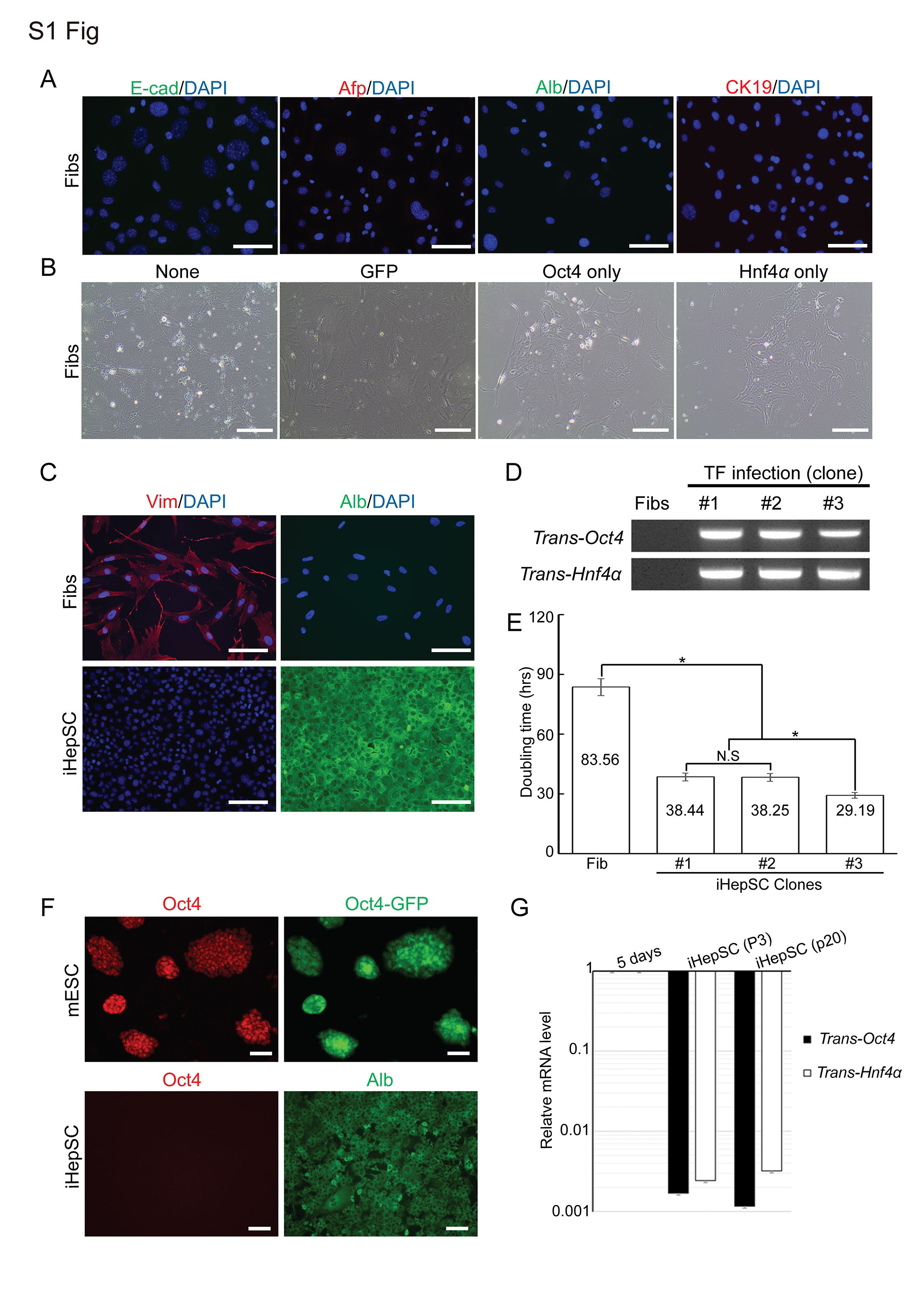

Supplement: S1 Fig — (A) Immunostaining analysis of mouse fibroblasts (Parental cells) with hepatic markers (E-cadherin: E-cad; α-fetoprotein: Afp; albumin: Alb; cytokeratin19: CK19). The nucleus was stained with DAPI. Scale bars: 150 μm. (B) Cell morphologies of TF induced fibroblasts (Fibs) at 21 days after transduction. Fibroblasts underwent proliferation arrest and cell death three weeks after transduction. Images indicated morphologies of no induction (none), mock-infection (GFP), single factor induction groups (Oct4 and Hnf4α only). Scale bars: 250 μm. (C) Immunostaining analysis of mouse fibroblasts and iHepSCs with hepatic marker (Alb) and fibroblast marker (Vimentin: Vim). The nucleus was stained with DAPI. Scale bars: 150 μm. (D) Genotyping of picked iHepSCs. Three iHepSC clones were mechanically isolated from infected plates. Genomic PCR analysis shows Oct4 and Hnf4α transgene insertion in the genome of three iHepSC clones (#1, #2, #3). Parental cells were used as negative controls. (E) Population doubling times of iHepSC clones (#1, #2 and #3) and parental cells (Fib). The values of the bar represent the mean of doubling time (mDT) of iHepSC clones and parental cells during 10 days. The experiment was carried out triplicate. *, P<0.05. N.S, not significance. (F) Immunostaining analysis of iHepSCs and mouse embryonic stem cells derived from OG2-ROSA transgenic mouse with pluripotency marker (Oct4) and hepatic marker (Alb). The cells were counterstained with DAPI. Scale bars: 150 μm. (G) Silencing of the exogenous Oct4 and Hnf4α genes in 2F iHepSCs. The expression levels were determined by qPCR using primer specific for transgenic transcripts. Transgenic expression levels of fibroblasts were compared with those in 5 days post-infection and on iHepSC clones at passage 3 and 20. Transcript levels were normalized to Gapdh. Error bars indicated standard errors from triplicate samples (n = 3). *, P<0.05. (TIF) [file pone.0221085.s001.tif]

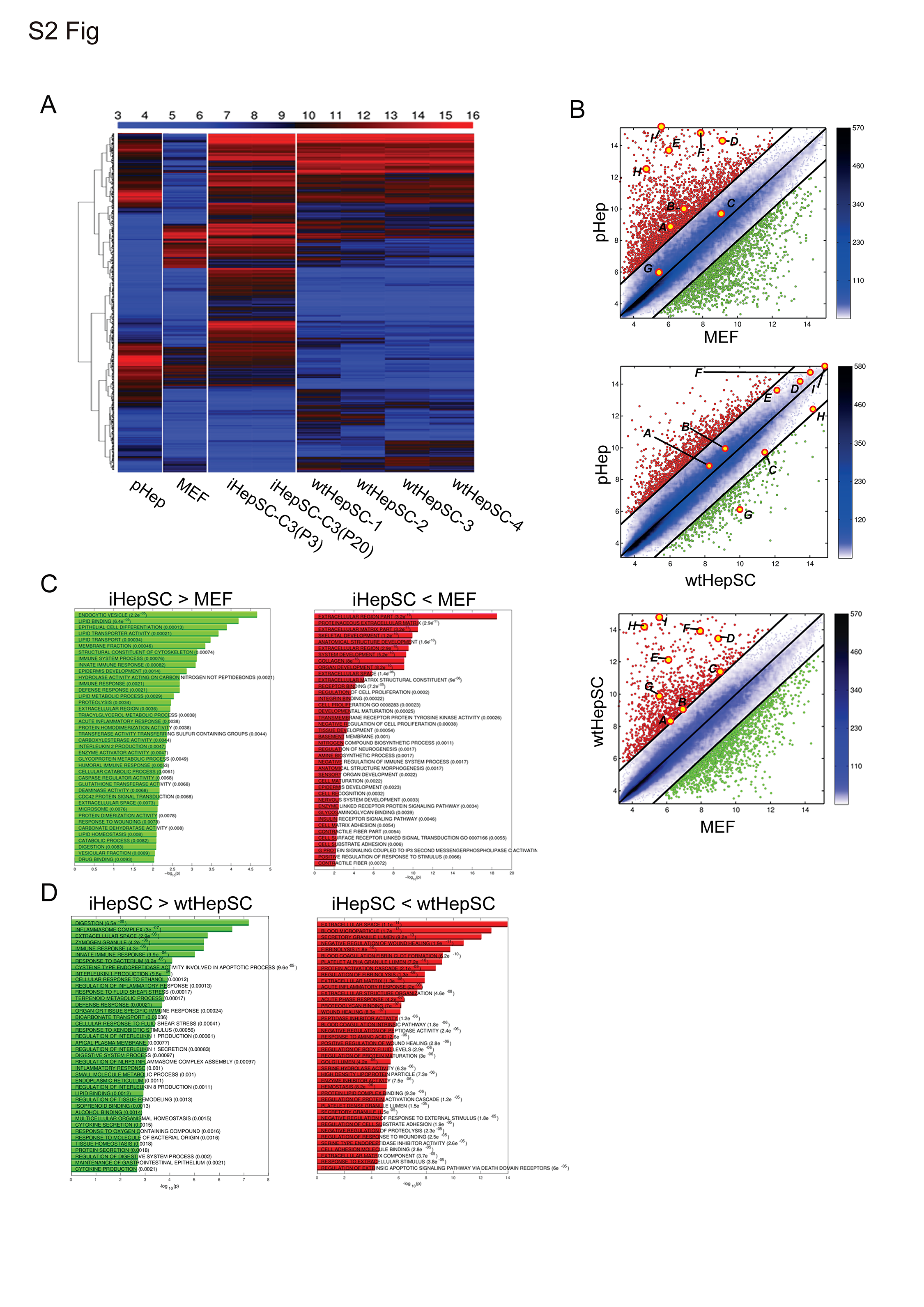

Supplement: S2 Fig — (A) Heatmap analysis of the global gene expression profiles of fibroblast (MEF), freshly isolated hepatocyte (pHep), iHepSC (P3), iHepSC (P20), and wild type HepSC (wtHepSC 1–4). The color bar in the top codifies the gene expression in log2 scale. (B) Pairwise scatter plots of samples; pHep vs wtHepSC (upper), pHep vs wtHepSC (middle), and wtHepSC vs MEF (lower). Hepatic markers were labelled as follow; (a) Gata6, (b) Sox9, (c) Ck19, (d) Ck18, (e) Hhex, (f) Ttr, (g) Epcam, (h) Afp, (i) Alb. (C) Functional enrichment analysis of parental cell and iHepSCs. On the left side of the panel, Plot bar of the -log10(p) of the significantly enriched terms of MEF-<-iHepSC-Log2(16). There are 366 differentially Down-regulated transcripts. On the right side of the panel, Plot bar of the -log10(p) of the significantly enriched terms of MEF->-iHepSC-Log2(16). There are 467 differentially Up-regulated transcripts. The longer the bar, the higher is the statistical significance of the enrichment. The p-values are written in parenthesis. (D) Functional enrichment analysis of wtHepSC and iHepSC. On the left side of the panel, Plot bar of the -log10(p) of the significantly enriched terms of wtHepSC-<-iHepSC-Log2(16). There are 239 differentially Down-regulated transcripts. On the right side of the panel, Plot bar of the -log10(p) of the significantly enriched terms of wtHepSC->-iHepSC-Log2(16). There are 218 differentially Up-regulated transcripts. The longer the bar, the higher is the statistical significance of the enrichment. The p-values are written in parenthesis. (TIF) [file pone.0221085.s002.tif]

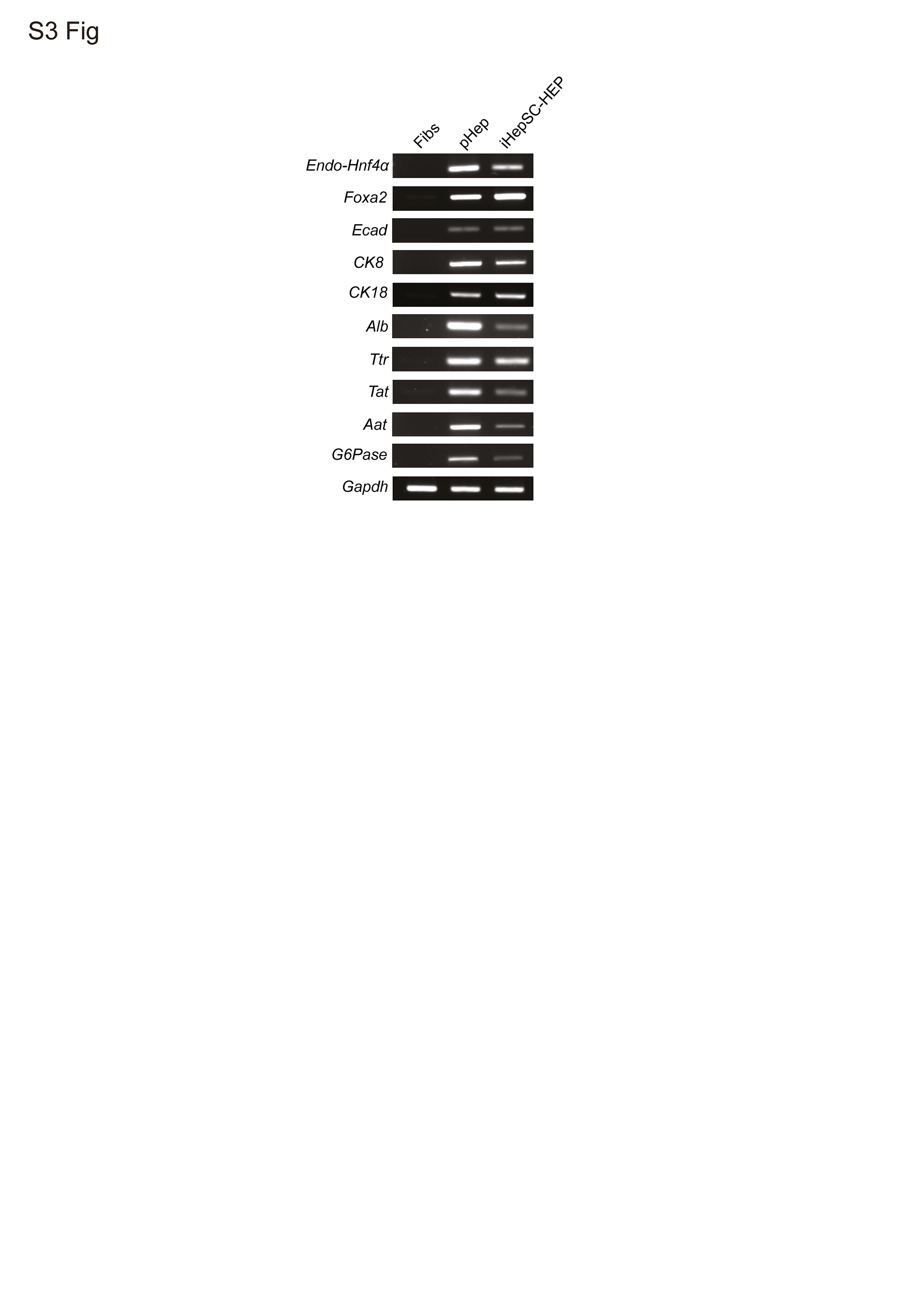

Supplement: S3 Fig — Gene analysis against hepatic stem cell and hepatocyte-specific markers, such as endogenous Hnf4α, Foxa2, E-cadherin (Ecad), Ck8, Ck18, Alb, transthyretin (Ttr), tyrosine aminotransferase (Tat), Alpha-1 antitrypsin (Aat) and glucose 6-phosphatase (G6Pase) of iHepSC-HEPs, pHeps, and Fibs. Fibs and pHeps were used as negative and positive controls. (TIF) [file pone.0221085.s003.tif]

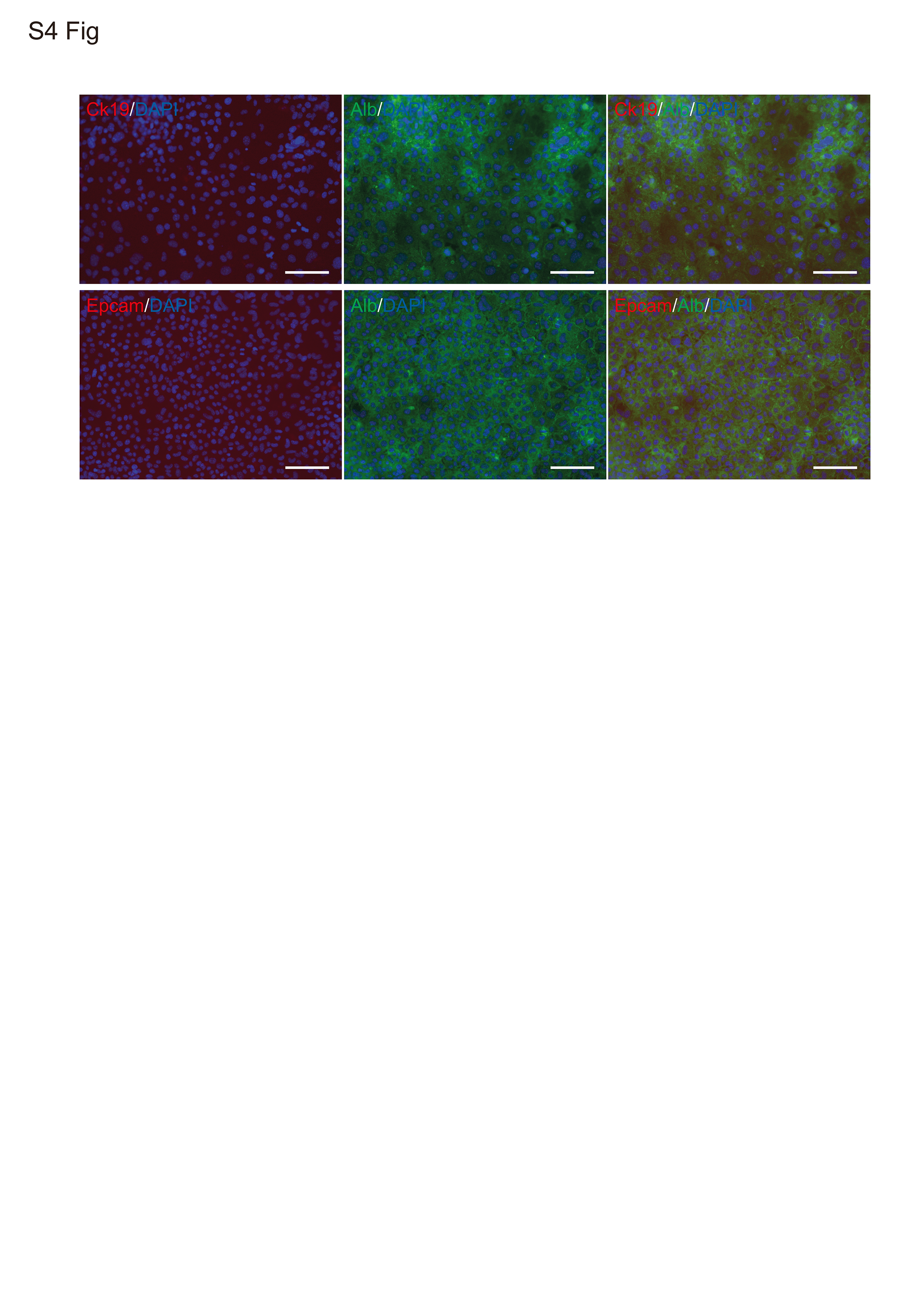

Supplement: S4 Fig — Immunostaining analysis revealed that iHepSC-HEPs negatively stained with hepatic stem cell markers (Epithelial cell adhesion molecule: Epcam) and cholangiocyte marker (Cytokeratin19: Ck19). The nucleus was stained with DAPI. Scale bar: 150 μm. (TIF) [file pone.0221085.s004.tif]

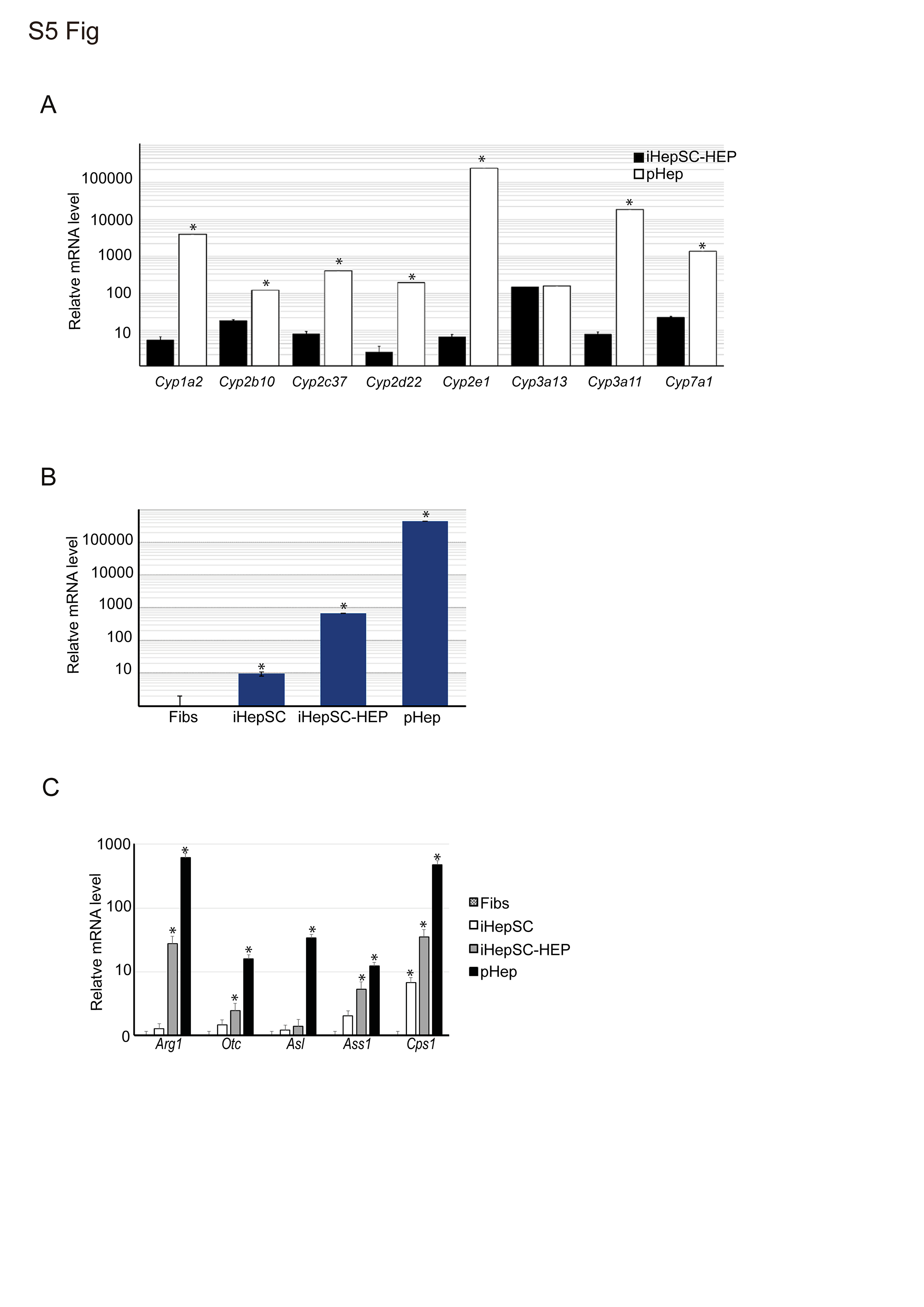

Supplement: S5 Fig — (A) Gene expression analysis against CYP family, such as Cyp1a2, Cyp2b10, Cyp2c37, Cyp2d22, Cyp2e1, Cyp3a11, Cyp3a13 and Cyp7a1 in iHepSC-HEP (black) and pHep (white) relative to parental cells. The transcriptional levels were normalized to a housekeeping gene (Gapdh) and represented in the logarithmic scale. Error bars indicated standard errors from triplicate samples (n = 3). (B-C) Gene expression analysis of Albumin (B) and urea cycle pathway (C) in iHepSC, iHepSC-HEP, and pHep relative to parental cells (fibs). The transcriptional levels were normalized to the housekeeping gene (Gapdh) and represented in the logarithmic scale. Error bars indicated standard errors from triplicate samples (n = 3). (TIF) [file pone.0221085.s005.tif]

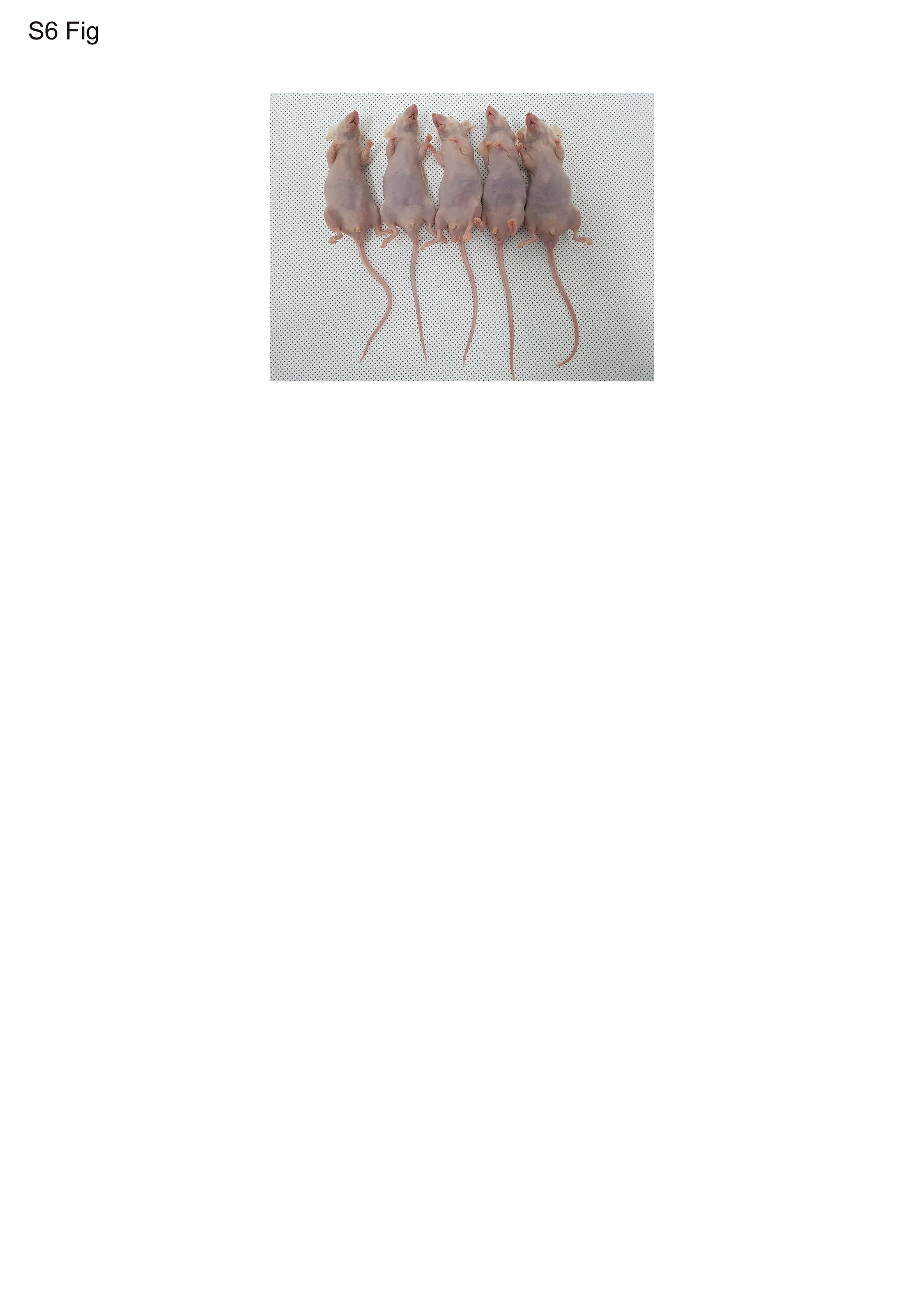

Supplement: S6 Fig — iHepSCs (2 x 10^6^ cells/mouse) were injected subcutaneously of the dorsal flank of athymic nude mice (n = 5). At 12 weeks after the subcutaneous injection, mice were sacrificed for analysis. No tumors were detected in recipient mice. (TIF) [file pone.0221085.s006.tif]

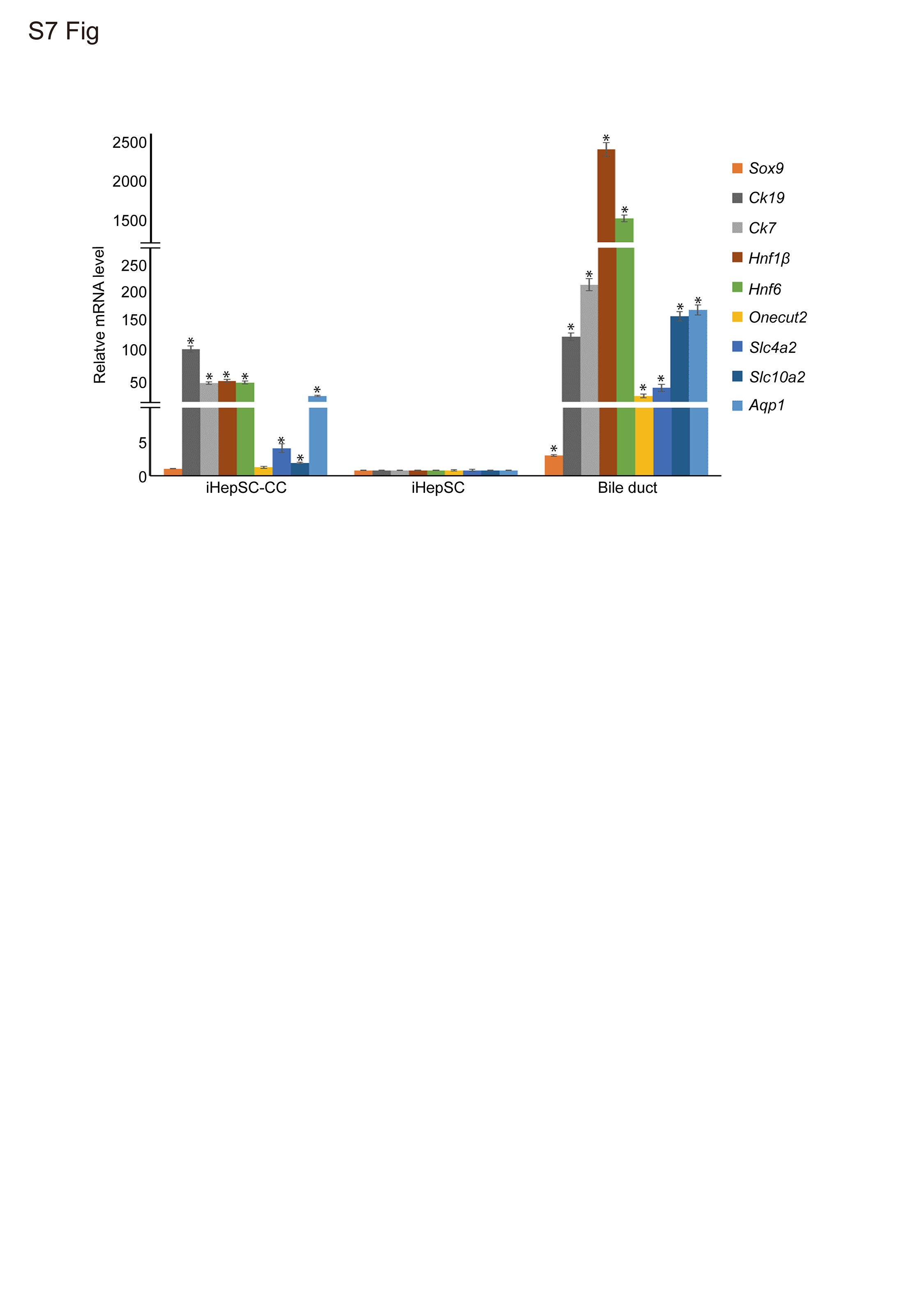

Supplement: S7 Fig — Gene expression analysis against cholangiocyte-specific markers including Sox9, Ck19, Ck7, Hnf1β, Hnf6, Onecut2, Slc4a2, Slc10a2, and Aqp1 in iHepSC-CC, iHepSC, and bile duct by qPCR. Mouse bile duct isolated from C57BL/6J mouse used as positive controls. The transcriptional levels were normalized to the housekeeping gene (Gapdh). Error bars indicated standard errors from triplicate samples (n = 3). *, P<0.05. (TIF) [file pone.0221085.s007.tif]

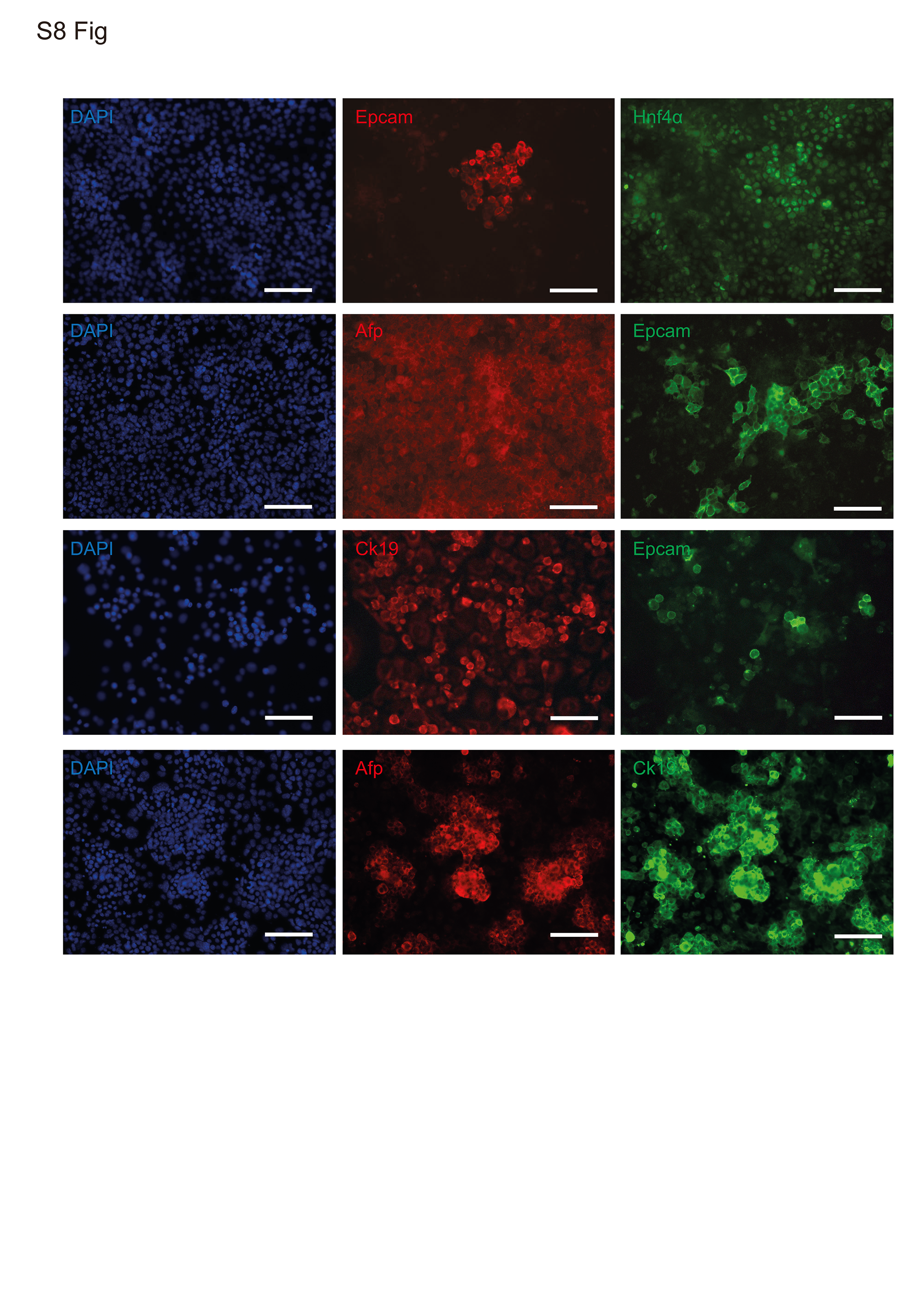

Supplement: S8 Fig — Multi-channel images of iHepSC stained with HepSC markers including Epithelial cell adhesion molecule: Epcam; hepatocyte nuclear factor 4 α: Hnf4; α-fetoprotein: Afp; Cytokeratin19: Ck19. The nucleus was stained with DAPI. Scale bar: 150 μm. (TIF) [file pone.0221085.s008.tif]

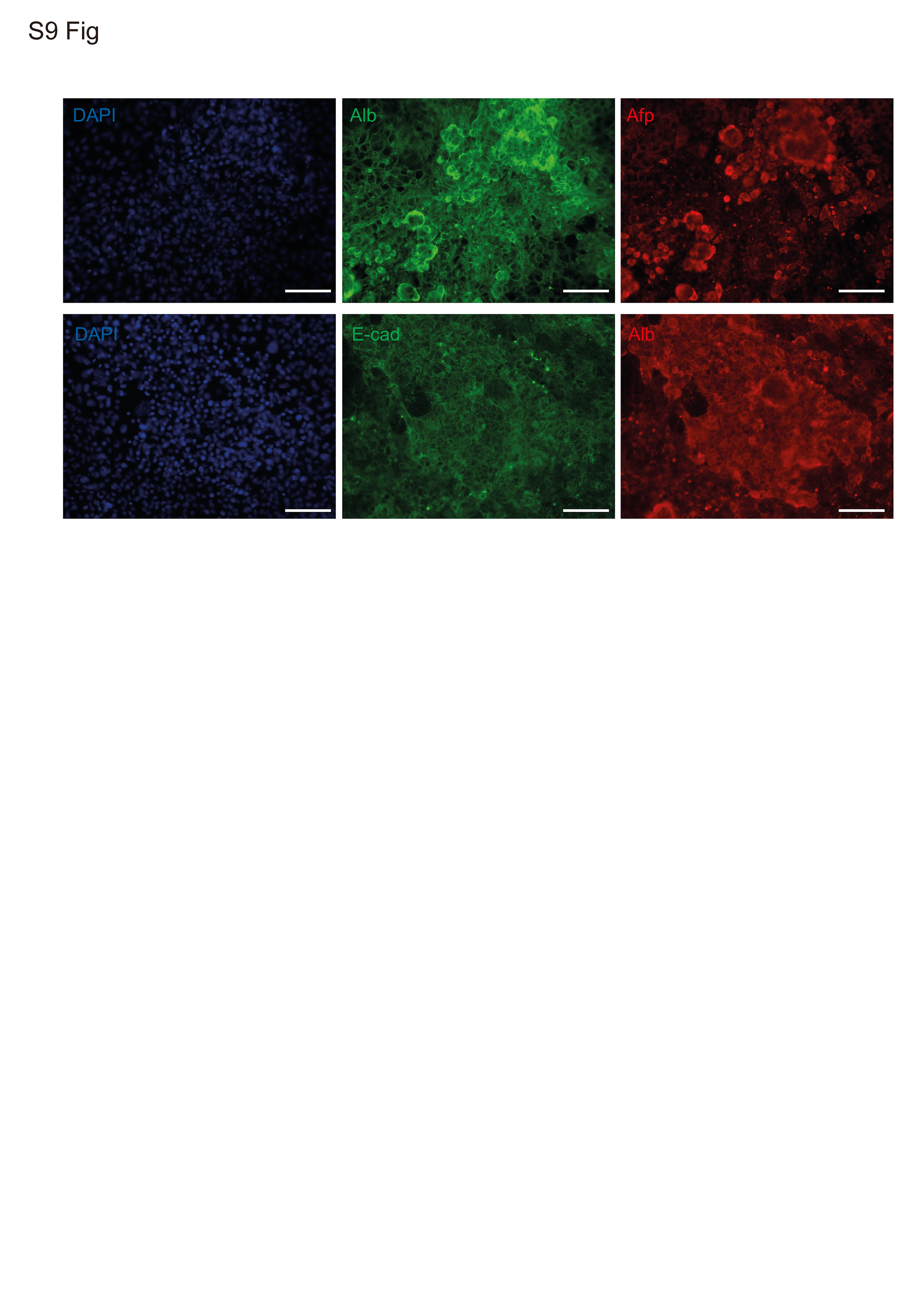

Supplement: S9 Fig — Multi-channel images of iHepSC-HEP stained with α-fetoprotein: Afp; Albumin: Alb; E-cadherin: E-cad. The nucleus was stained with DAPI. Scale bar: 150 μm. (TIF) [file pone.0221085.s009.tif]

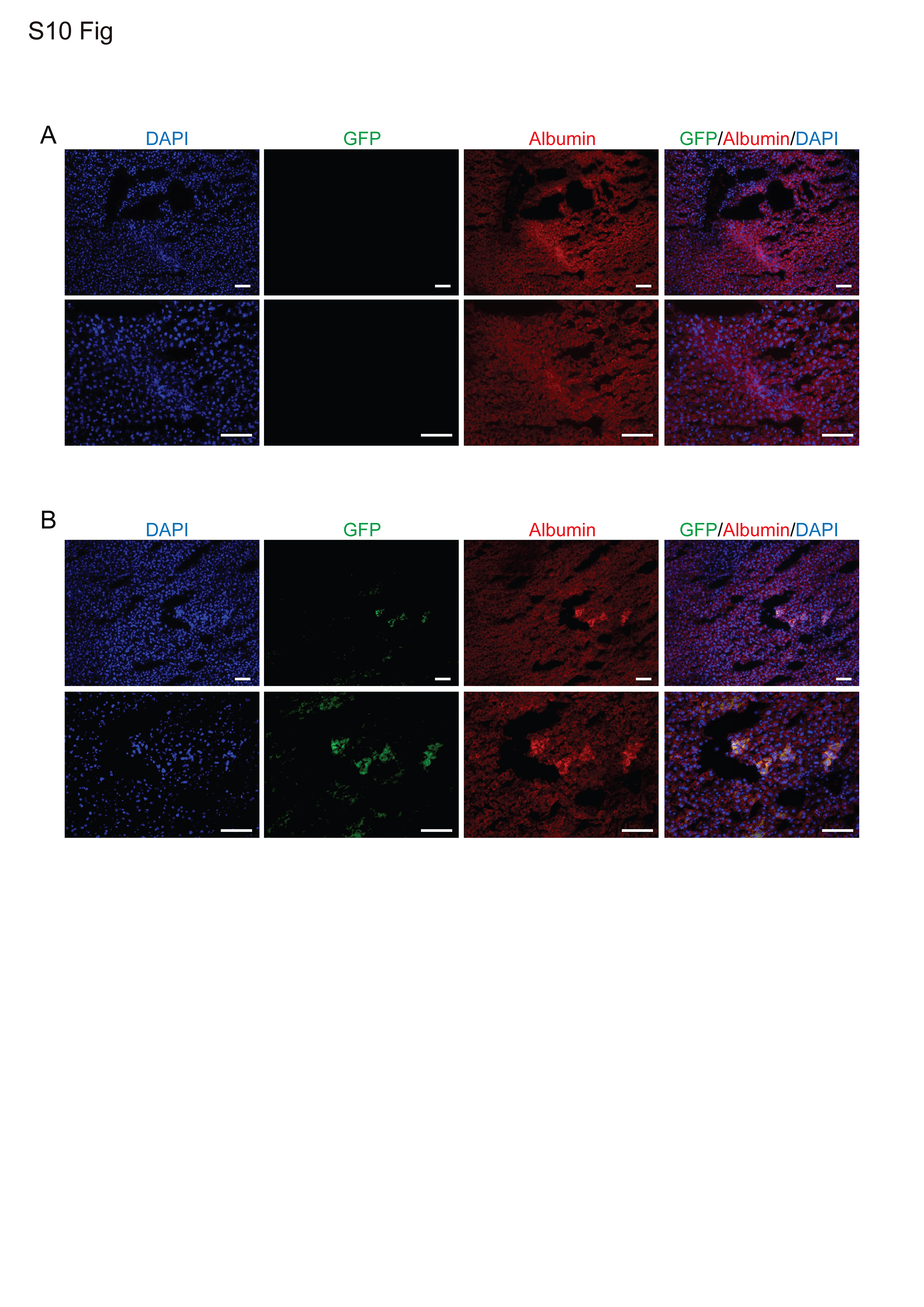

Supplement: S10 Fig — Multi-channel images of iHepSCs stained with anti-GFP (Green) and anti-albumin (Red) at liver sections of both PBS injected mine (A) and iHepSCs transplanted mice (B). The nucleus was stained with DAPI. Scale bar: 150 μm. (TIF) [file pone.0221085.s010.tif]

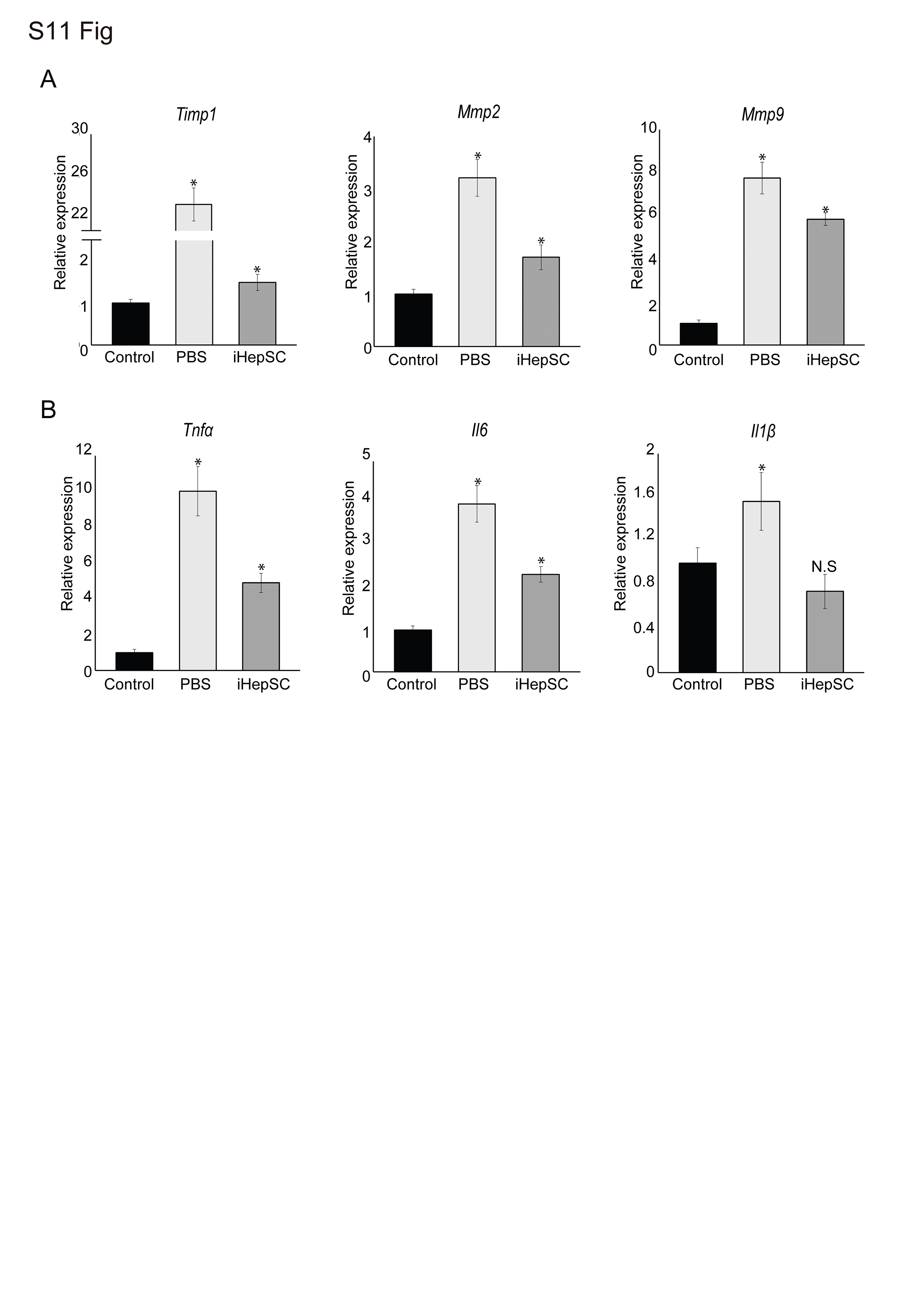

Supplement: S11 Fig — (A) The transcription levels of genes related to ECM modulation including, Tissue inhibitor of metalloprotease protein1: Timp1; Matrix metalloproteinases2: Mmp2; Matrix metalloproteinase9: Mmp9 in liver tissues by qPCR. The transcriptional levels were normalized by the housekeeping gene (Gapdh). Error bars indicated standard errors from triplicate samples (n = 3). *, P<0.05. (B) The transcription levels of genes involved in an inflammatory response including, Tumor necrosis factor-alpha: Tnfα; Interleukin6: Il6; Interleukin1 beta: Il1β in liver tissues by qPCR. The transcriptional levels were normalized by the housekeeping gene (Gapdh). Error bars indicated standard errors from triplicate samples (n = 3). *, P<0.05. (TIF) [file pone.0221085.s011.tif]

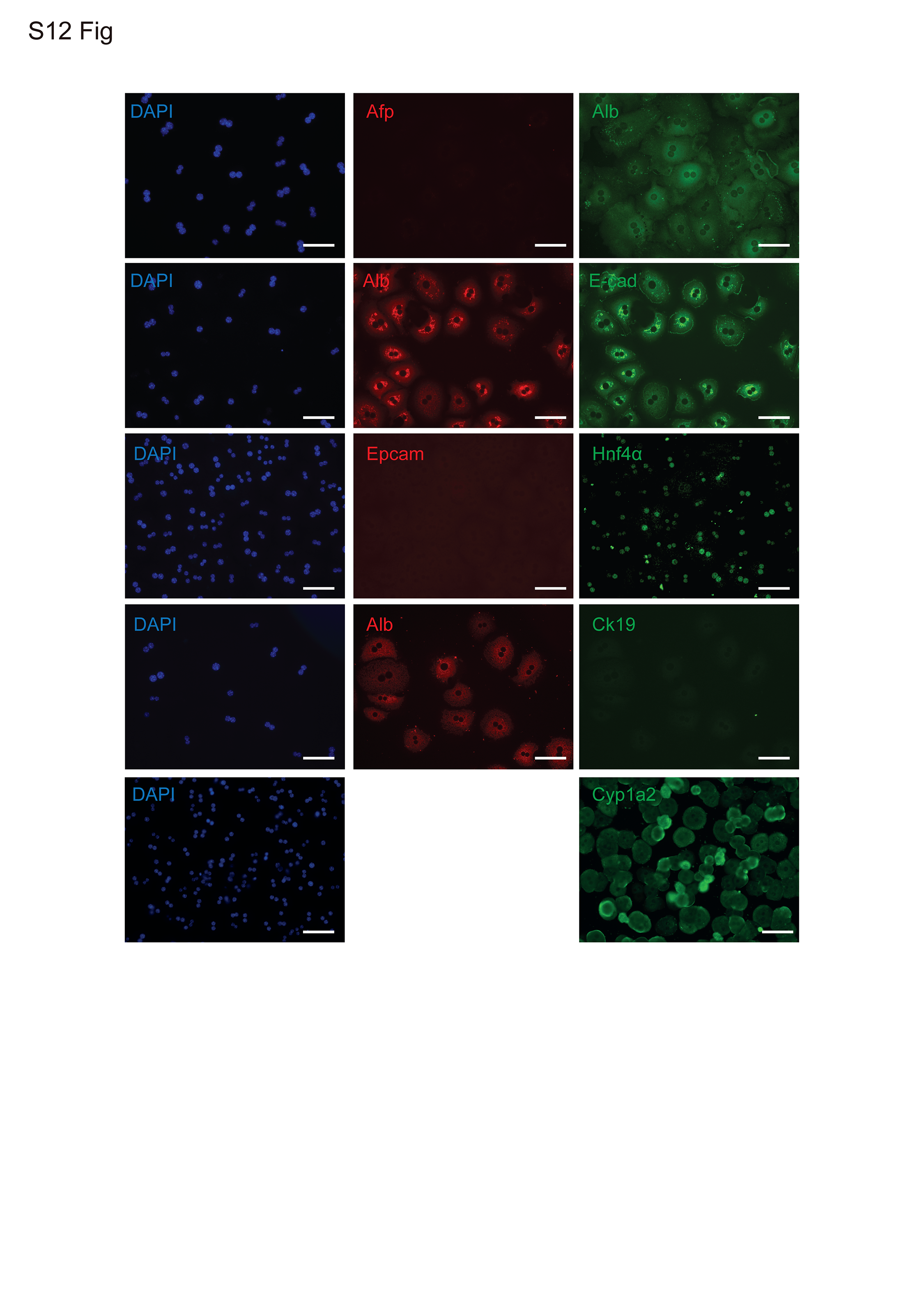

Supplement: S12 Fig — Immunostaining images of primary hepatocytes stained with hepatic markers including, Afp, Alb, E-cad, Hnf4α, Ck19, and Cyp1a2. The nucleus was stained with DAPI. Scale bar: 150 μm. (TIF) [file pone.0221085.s012.tif]

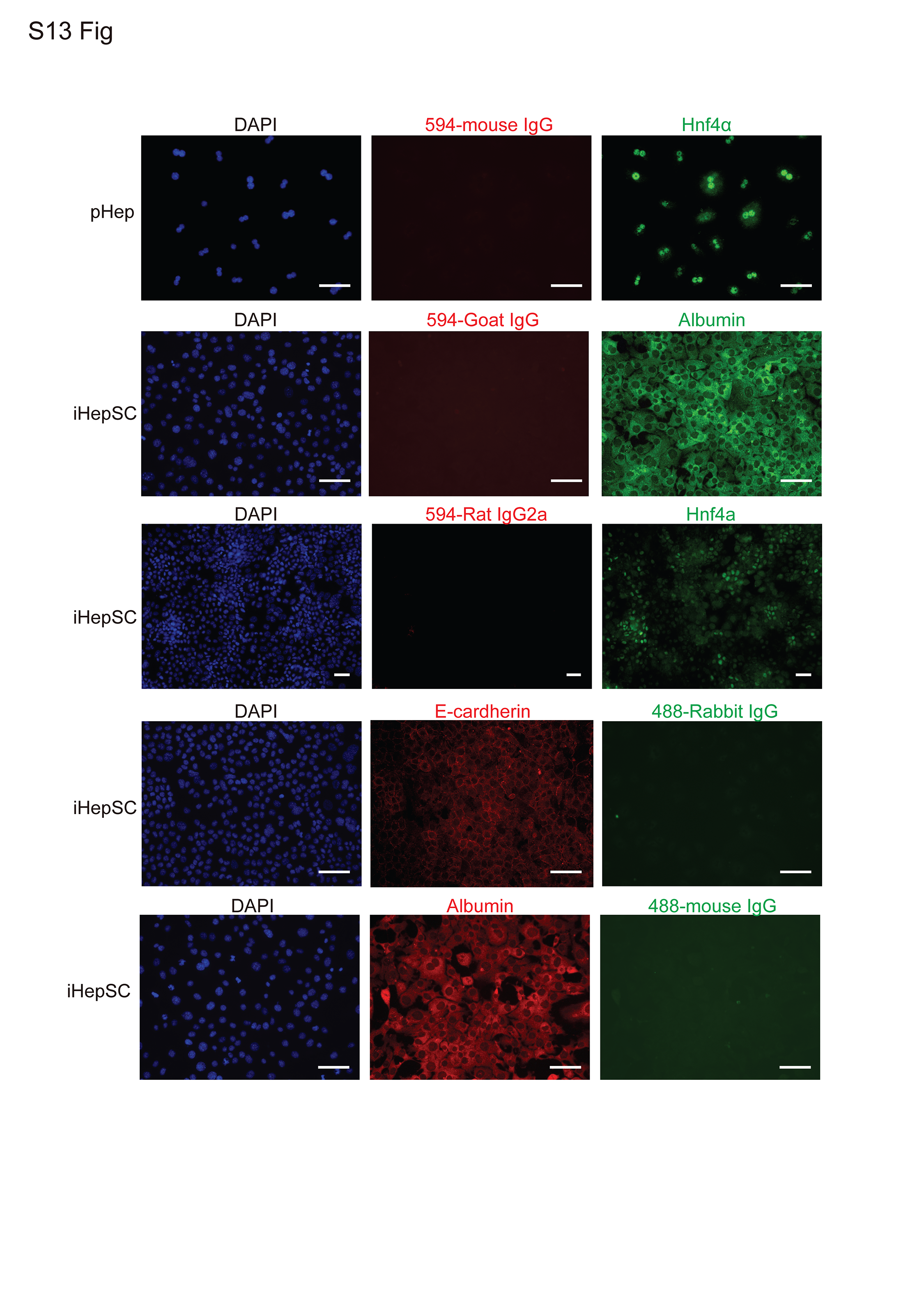

Supplement: S13 Fig — Immunostaining images of primary hepatocytes and iHepSC-HEP stained with hepatic markers including, Hnf4α, E-cad, and alb. The nucleus was stained with DAPI. Mouse IgG, Goat IgG, Rat IgG2a, Rabbit IgG, and Mouse IgG antibodies were used as isotype controls. Scale bar: 150 μm. (TIF) [file pone.0221085.s013.tif]
