# Supplementary material for: Oct4 and Hnf4α-induced hepatic stem cells ameliorate chronic liver injury in liver fibrosis model
Source: PLoS One. 2019 Aug 12;14(8):e0221085. doi: 10.1371/journal.pone.0221085 (PMC6690533; doi:10.1371/journal.pone.0221085)
Supplement: S1 Table — (DOCX) [file pone.0221085.s014.docx]

**S1 Table. Differentially expressed gene analysis among liver metabolic activity of iHepSC VS. Parental cells (MEF) and primary hepatocyte (pHep) VS. MEF.**

Global gene expression profiles of iHepSC, primary hepatocyte (pHep) and parental cells (MEF) were analyzed by using Affymetrix GeneChip Mouse 430 2.0 Array. Average expression levels of each listed gene in iHepSCs were divided by the expression level of that gene in MEF to calculate the ratio of iHepSC/MEF. The ratio of pHep/MEF was calculated via dividing the expression level in pHep by the expression level in MEF. The lists of liver metabolic activity were extracted (Values of P<0.05). Genes that exhibited significantly different expression levels among genes involved in glucose (A), drug metabolism (B), coagulation (C), fatty acid, cholesterol, bile acid metabolism (D) and secretory protein (E) were selected.

(A) Glucose metabolism

|  | iHepSC/MEF | pHep/MEF |
| --- | --- | --- |
| aldob | 25.5 | 446.8 |
| pklr | 2.6 | 14.9 |
| ppargc1a | 2.0 | 3.5 |
| slc25a10 | 2.8 | 2.9 |
| ugdh | 3.0 | 1.2 |

(B) drug metabolism

|  | iHepSC/MEF | pHep/MEF |
| --- | --- | --- |
| gnpnat1 | 3.8 | 2.7 |
| gsta1 | 16.7 | 1.7 |
| gsta2 | 2.2 | 1.1 |
| gsta3 | 12.5 | 135.4 |
| gstm1 | 4.7 | 11.5 |
| gstm3 | 1.6 | 39.1 |
| gstm7 | 1.6 | 7.6 |
| gstp1 | 1.7 | 1.6 |
| maob | 2.5 | 4.2 |
| sult1a1 | 319.0 | 856.8 |
| sult1b1 | 52.6 | 11.7 |
| sult1c2 | 152.9 | 6.2 |
| ugt1a9 | 5.0 | 2.5 |
| ugt2b34 | 6464.4 | 2919.1 |
| ugt2b35 | 220.2 | 83.9 |
| ugt2b36 | 57.4 | 94.6 |
| ugt2b37 | 4.3 | 26.3 |
| ugt2b38 | 5.4 | 2.2 |
| ugt2b5 | 782.8 | 2965.3 |

(C) Coagulation

|  | iHepSC/MEF | pHep/MEF |
| --- | --- | --- |
| c3 | 375.0 | 1137.1 |
| c4bp | 39.8 | 55.5 |
| c9 | 188.1 | 61.0 |
| f11r | 10.6 | 9.1 |
| f2 | 2.7 | 296.0 |
| f2rl1 | 2.6 | 2.4 |
| f8a | 1.4 | 1.5 |
| fga | 6.3 | 706.0 |
| fgb | 123.3 | 15770.8 |
| serpinf2 | 1.8 | 162.4 |

(D) fatty acid cholesterol bile acid metabolism

|  | iHepSC/MEF | pHep/MEF |
| --- | --- | --- |
| abca2 | 2.9 | 2.8 |
| acox1 | 2.1 | 3.4 |
| acox2 | 106.0 | 52.0 |
| acsl1 | 2.6 | 8.8 |
| acsl3 | 2.3 | 1.3 |
| acsl4 | 2.5 | 3.3 |
| acsm2 | 1.9 | 1.2 |
| acss2 | 3.9 | 9.1 |
| cd36 | 20.2 | 29.0 |
| fabp1 | 32.4 | 101.8 |
| got2 | 2.2 | 4.8 |
| ldlr | 1.3 | 3.9 |
| ucp2 | 19.0 | 2.8 |

(E) secretory protein

|  | iHepSC/MEF | pHep/MEF |
| --- | --- | --- |
| agt | 141.6 | 2841.8 |
| alb1 | 1206.0 | 1420.7 |
| apoa1 | 6342.4 | 5638.5 |
| apob | 366.1 | 397.2 |
| apoc1 | 7.1 | 124.8 |
| apoc2 | 102.7 | 514.8 |
| apoc3 | 20.5 | 288.1 |
| cp | 93.8 | 240.1 |
| gc | 92.5 | 3494.6 |
| hp | 940.4 | 1315.2 |
| hpx | 150.9 | 288.5 |
| rbp4 | 3.3 | 62.8 |
| serpina7 | 33.5 | 1061.5 |
| ttr | 13433.6 | 14088.8 |
