# Supplementary material for: Oct4 and Hnf4α-induced hepatic stem cells ameliorate chronic liver injury in liver fibrosis model
Source: PLoS One. 2019 Aug 12;14(8):e0221085. doi: 10.1371/journal.pone.0221085 (PMC6690533; doi:10.1371/journal.pone.0221085)
Supplement: S2 Table — (DOCX) [file pone.0221085.s015.docx]

**S2 Table. Lists of primer sequences**

**List of primer sequences for RT-PCR analysis**

| **Gene** | **ACCESSION Number** | **Sequence** |
| --- | --- | --- |
| *Foxa2* | NM_010446.2 | F: CACCTGAGTCCGAGTCTGAG  R: AAGGAGAGAGAGTGGCGGAT |
| *Hnf4α* | NM_008261.2 | F: AGGCAATGACTACATCGTCCC  R: CAGACCCTCCGAGAAGCATC |
| *E-cad* | NM_009864.2 | F: GCAGGTCTCCTCATGGCTTTG  R: TTGGATTCAGAGGCAGGGTCG |
| *Epcam* | NM_008532.2 | F: GGTGAATGCCAGTGTACTT  R: CAATGATGATCCAGTAGGTCC |
| *Alb* | NM_009654.3 | F: TGAAGTTGCCAGAAGACATCC  R: CAAGTTCCGCCCTGTCATCTG |
| *Ttr* | NM_013697.5 | F: GCTTCCCTTCGACTCTTCCTC  R: GCCAAGTGTCTTCCAGTACGA |
| *Afp* | NM_007423.4 | F: GCAGGATGGGGAAAAAGTCA  R: CCTAAGGTCTGGTAGAGAGCG |
| *Aat* | NM_009243.4 | F: GACCAAGACACAGTTTTCGC  R: ATCTGGGCTAACCTTCTGCG |
| *Tat* | NM_146214.3 | F: ATCGGCTACCTATCCAGTCG  R: GCCACTGCCAAAATCTTCTGA |
| *CK8* | NM_031170.2 | F: AGAAGGATGTGGACGAAGCA  R: ATCTCTGTCTTTGTGCGGCG |
| *CK18* | NM_010664.2 | F: ATGAAGAGGAAGTCCAAGGTC  R: GTTCTCCAAGTTGATGTTCTG |
| *G6P* | NM_008061.3 | F: TCAACCTCGTCTTCAAGTGGATT  R: CACAGCAATGCCTGACAAGA |
| *Gapdh* | NM_001289726.1 | F: ACGACCCCTTCATTGACCTCAACT  R: ATATTTCTCGTGGTTCACACCCAT |

**Genomic PCR primer for *Oct4* and *Hnf4α* transgene**

| **Gene** | **Forward primer (5'-3')** | **Reverse primer (5'-3')** |
| --- | --- | --- |
| SF*-mOct4* | AGACCACCATCTGTCGCTTC | AAGGCATTAAAGCAGCGTATCCA |
| SF*-mHnf4α* | AGGCAATGACTACATCGTCCC | AAGGCATTAAAGCAGCGTATCCA |

**qRT**–**PCR primer for *Oct4* and *Hnf4α* transgene**

| **Gene** | **Forward primer (5'-3')** | **Reverse primer (5'-3')** |
| --- | --- | --- |
| SF*-mOct4* | ACTCTACTCAGTCCCTTTTCC | AAGGCATTAAAGCAGCGTATCCA |
| SF*-mHnf4α* | CATCGTCAAGCCTCCCTCTG | AAGGCATTAAAGCAGCGTATCCA |

**List of primer sequnces for qPCR analysis**

| **Gene** | **ACCESSION Number** | **Sequence** |
| --- | --- | --- |
| *Foxa2* | NM_010446.2 | F: AACATGAACTCGATGAGCCC  R: GATGTACGAGTAGGGAGGTTTG |
| *Gata6* | NM_010258.3 | F: TCTACAGCAAGATGAATGGCC  R: CTCACCCTCAGCATTTCTACG |
| *Hnf4a* | NM_008261.2 | F: CTAACACGATGCCCTCTCAC  R: GCAGGAGCTTGTAGGATTCAG |
| *Epcam* | NM_008532.2 | F: TGGTGTCATTAGCAGTCATCG  R: TCAGTTCAGCACTCAGCAC |
| *Ck18* | NM_010664.2 | F: ACACCAACATCACAAGGCTG  R: TTCCACAGTCAATCCAGAGC |
| *Ck19* | NM_008471.3 | F: GTTCTCAGACCTGCGTCC  R: TGACCCAATGCGTACTGAAC |
| *Cyp1a2* | NM_009993.3 | F: ATAACTTCGTGCTGTTTCTGC  R: ACCGCCATTGTCTTTGTAGT |
| *Cyp1b1* | NM_009994.1 | F: ATTCTCAGTGGGCAAACGG  R: GGATTCTAAACGACTTGGGCT |
| *Cyp2b10* | NM_009999.4 | F: CTGTCGTTGAGCCAACCTTC  R: TCCGCAGTTCCTCCACTAAA |
| *Cyp2c37* | NM_010001.2 | F: TGTGGAGGAACTTAGGAAAACC  R: AGGGCTGCTCAGAATCTTTGT |
| *Cyp2d22* | NM_001163472.1 | F: GCCTTCATGCCATTCTCAGC  R: CAGAGCCCTAAAGACGCC |
| *Cyp2e1* | NM_021282.2 | F: GGAATGGGGAAACAGGGTAAT  R: GCACAGCCAATCAGAAAGGT |
| *Cyp3a11* | NM_007818.3 | F: TGGGACTCGTAAACATGAACTT  R: TTGACCATCAAACAACCCCC |
| *Cyp3a13* | NM_007819.4 | F: GGGGACGATTCTTGCTTACC  R: AAATACCCACTGGACCAAAGC |
| *Cyp7a1* | NM_007824.2 | F: AACGATACACTCTCCACCTTTG  R: CTGCTTTCATTGCTTCAGGG |
| *Arg1* | NM_007482.3 | F: AAGAATGGAAGAGTCAGTGTGG  R: AGTGTTGATGTCAGTGTGAGC |
| *Otc* | NM_008769.4 | F: ATGCAGCTCTTAGAAAGGGTC  R: TGTACTTGACTTTGGACTGGC |
| *Asl* | NM_133768.5 | F: TCAATGTCTTGCCACTGGG  R: ACAGGAACTCAGCCACAAAG |
| *Ass1* | NM_007494.3 | F: CCACGCTCATTTAGACATAGAGG  R: AACAAATTCACATTCAGGGCTG |
| *Cps1* | NM_001080809.2 | F: AAGTAGAGATGGACGCTGTTG  R: CTTGGCTGATGGTCTGTGTAG |
| *Sox9* | NM_011448.4 | F: AAGAGACCCTTCGTGGAGGA  R: CTCCGCTTGTCCGTTCTTCA |
| *Ck7* | NM_033073.3 | F: CCACAATTGAGCACATACAACC  R: TCAGACTATCTTCCAGGCTCG |
| *Hnf1β* | NM_009330.3 | F: AGCACCTTGACGAATATCCAC  R: AGCCACACTGTTAATGACCG |
| *Hnf6* | NM_008262.3 | F: AGCAAACTCAAGTCGGGTC  R: GTGTTGCCTCTGTCCTTCC |
| *Onecut2* | NM_194268.2 | F: ATTCTCAGTGGGCAAACGG  R: GGATTCTAAACGACTTGGGCT |
| *Slc4a2* | NM_009207.3 | F: TCTTTGCCTTCCTCATATCACTC  R: GTTGCCCATGTCATATTGCTG |
| *Slc10a2* | NM_011388.3 | F: TGTGACAAAGATCCAAAGATATGTG  R: CTTGTGACCAATCTGACTATGAATAC |
| *Aqp1* | NM_007472.2 | F: CTGGCGATTGACTACACTGG  R: AAGTCATAGATGAGCACTGCC |
| *Tnfα* | NM_013693.3 | F: AGACCCTCACACTCAGATCA  R: TGTCTTTGAGATCCATGCCG |
| *Il1β* | NM_008361.4 | F: ACGGACCCCAAAAGATGAAG  R: TTCTCCACAGCCACAATGAG |
| *Il6* | NM_031168.2 | F: GATAAGCTGGAGTCACAGAAGG  R: GGAATGTCCACAAACTGATATGC |
| *Timp1* | NM_011593.2 | F: CTCAAAGACCTATAGTGCTGGC  R: CAAAGTGACGGCTCTGGTAG |
| *Mmp2* | NM_008610.3 | F: ACCAAGAACTTCCGATTATCCC  R: CAGTACCAGTGTCAGTATCAGC |
| *Mmp9* | NM_013599.4 | F: GATCCCCAGAGCGTCATTC  R: CCACCTTGTTCACCTCATTTTG |
| *Gapdh* | NM_001289726.1 | F: TGCCCCCATGTTTGTGAT  R: TGTGGTCATGAGCCCTTC |
