# Supplementary material for: Oct4 and Hnf4α-induced hepatic stem cells ameliorate chronic liver injury in liver fibrosis model
Source: PLoS One. 2019 Aug 12;14(8):e0221085. doi: 10.1371/journal.pone.0221085 (PMC6690533; doi:10.1371/journal.pone.0221085)
Supplement: S3 Table — (DOCX) [file pone.0221085.s016.docx]

**S3 Table. List of primary antibodies**

|  | **Antigen** | **Source** | **Isotype** | **Dilution** |
| --- | --- | --- | --- | --- |
| Primary antibody | Afp | R&D systems | Mouse IgG | 1:400 |
|  | Alb | Abcam | Goat IgG | 1:400 |
|  | Ck19 | DSHB | Rat IgG2a | 1:200 |
|  | Ck7 | Abcam | Mouse IgG | 1:200 |
|  | Cyp1a2 | Abcam | Rabbit IgG | 1:400 |
|  | E-cad | Abcam | Mouse IgG | 1:200 |
|  | Epcam | DSHB | Rat IgG2a | 1:100 |
|  | GFP | Abcam | chicken IgY | 1:1000 |
|  | Hnf4a | Santa cruz | Rabbit IgG | 1:100 |
|  | Oct4 | Santa cruz | Goat IgG | 1:400 |
|  | Vimentin | Abcam | Rabbit IgG | 1:400 |
